# Occupancy maps of 208 chromatin-associated proteins in one human cell type

**DOI:** 10.1038/s41586-020-2023-4

**Published:** 2020-07-29

**Authors:** E. Christopher Partridge, Surya B. Chhetri, Jeremy W. Prokop, Ryne C. Ramaker, Camden S. Jansen, Say-Tar Goh, Mark Mackiewicz, Kimberly M. Newberry, Laurel A. Brandsmeier, Sarah K. Meadows, C. Luke Messer, Andrew A. Hardigan, Candice J. Coppola, Emma C. Dean, Shan Jiang, Daniel Savic, Ali Mortazavi, Barbara J. Wold, Richard M. Myers, Eric M. Mendenhall

**Affiliations:** 1 HudsonAlpha Institute for Biotechnology, Huntsville, AL USA; 2 Department of Biological Sciences, The University of Alabama in Huntsville, Huntsville, AL USA; 3 Department of Pediatrics and Human Development, College of Human Medicine, Michigan State University, Grand Rapids, MI USA; 4 Department of Genetics, University of Alabama at Birmingham, Birmingham, AL USA; 5 Department of Developmental and Cell Biology, University of California Irvine, Irvine, CA USA; 6 Division of Biology, California Institute of Technology, Pasadena, CA USA; 7 Department of Pathology, University of Alabama at Birmingham, Birmingham, AL USA; 8 Pharmaceutical Sciences Department, St Jude Children’s Research Hospital, Memphis, TN USA; 9 Present Address: Department of Biomedical Engineering, Johns Hopkins University, Baltimore, MA USA

**Keywords:** DNA, Epigenomics, Gene regulation, Transcriptomics

## Abstract

Transcription factors are DNA-binding proteins that have key roles in gene regulation^1,2^. Genome-wide occupancy maps of transcriptional regulators are important for understanding gene regulation and its effects on diverse biological processes^3–6^. However, only a minority of the more than 1,600 transcription factors encoded in the human genome has been assayed. Here we present, as part of the ENCODE (Encyclopedia of DNA Elements) project, data and analyses from chromatin immunoprecipitation followed by high-throughput sequencing (ChIP–seq) experiments using the human HepG2 cell line for 208 chromatin-associated proteins (CAPs). These comprise 171 transcription factors and 37 transcriptional cofactors and chromatin regulator proteins, and represent nearly one-quarter of CAPs expressed in HepG2 cells. The binding profiles of these CAPs form major groups associated predominantly with promoters or enhancers, or with both. We confirm and expand the current catalogue of DNA sequence motifs for transcription factors, and describe motifs that correspond to other transcription factors that are co-enriched with the primary ChIP target. For example, FOX family motifs are enriched in ChIP–seq peaks of 37 other CAPs. We show that motif content and occupancy patterns can distinguish between promoters and enhancers. This catalogue reveals high-occupancy target regions at which many CAPs associate, although each contains motifs for only a minority of the numerous associated transcription factors. These analyses provide a more complete overview of the gene regulatory networks that define this cell type, and demonstrate the usefulness of the large-scale production efforts of the ENCODE Consortium.

## Abstract (web summary)

ChIP–seq and CETCh–seq data are used to analyse binding maps for 208 transcription factors and other chromatin-associated proteins in a single human cell type, providing a comprehensive catalogue of the transcription factor landscape and gene regulatory networks in these cells.

## Main

There are an estimated 1,639 transcription factors (TFs) in the human genome^2^, and up to 2,500 CAPs when we include transcriptional cofactors, RNA polymerase-associated proteins, histone-binding regulators, and chromatin-modifying enzymes^1,7^. A typical TF binds to a short DNA sequence motif, and, in vivo, some TFs exhibit additional chromosomal occupancy mediated by their interactions with other CAPs^8–10^. CAPs are vital for orchestrating cell type- and cell state-specific gene regulation, including the temporal coordination of gene expression in developmental processes, environmental responses, and disease states^3–6,11–13^.

Identifying genomic regions with which a TF is physically associated, referred to as TF binding sites (TFBSs), is an important step towards understanding its biological roles. The most common genome-wide assay for identifying TFBSs is ChIP–seq^14–16^. In addition to highlighting potentially active regulatory DNA elements by direct measurement, ChIP–seq data can define DNA sequence motifs that can be used, often in conjunction with expression data and chromatin accessibility maps, to infer likely binding events in other cellular contexts without performing direct assays. Although motifs identified by ChIP–seq are often representative of direct binding, this is not always the case, as co-occurrence of other TFs could lead to the enrichment of their motifs. Furthermore, the ChIP–seq method identifies both protein–DNA and, indirectly, protein–protein interactions, such that indirect and even long-distance interactions (for example, looping of distal elements) can be captured as ChIP–seq enrichments.

A long-term goal is comprehensive mapping of all CAPs in all cell types, but a more immediate aspiration is to create a catalogue of all CAPs expressed in a single cell type. The resulting consolidation of hundreds of genome-wide maps for a single cellular context promises insights into CAP networks that are otherwise not possible. Such comprehensive data will also provide the backdrop for understanding large-scale functional element assays, and should improve the ability to infer TFBSs in other cell types that are less amenable to direct measurements.

**Fig. 1:**
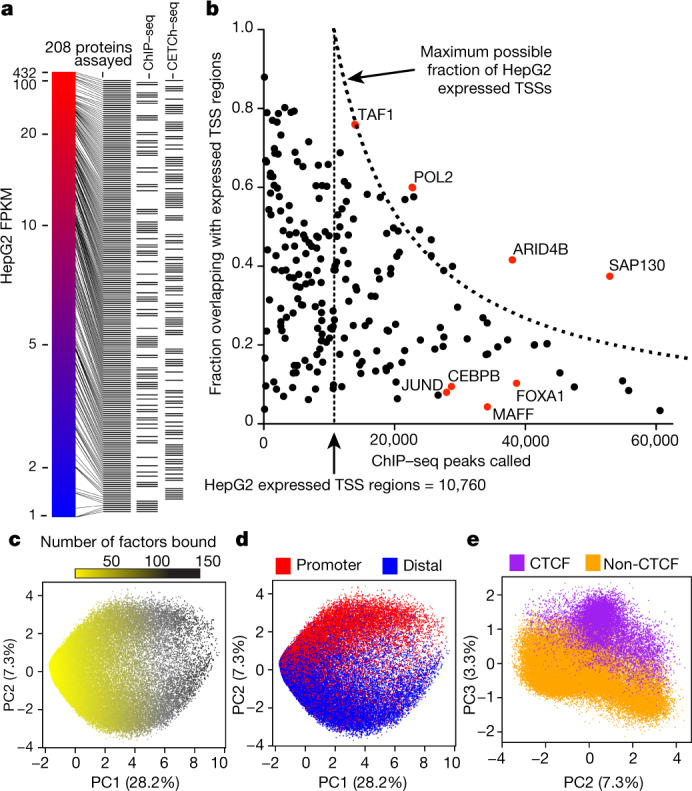
Overview and analysis of HepG2 data sets. **a**, The 208 chromatin-associated factors assayed in HepG2 cells, organized by expression (FPKM), and denoting whether the factors were assayed by ChIP–seq or CETCh–seq. **b**, Scatter plot of all 208 factors, showing broad distribution of fraction of called peaks at expressed TSSs (±3 kb from TSS) against total peak number; points beyond the maximum possible fraction are possible owing to multiple peaks at single TSS regions. **c**, Plot showing PCA of genomic segments (*n* = 282,105) with more than two factors bound, highlighting the separation on the basis of the number of factors bound. **d**, Same plot as in **c** showing promoter versus distal location. **e**, Same plot as in **c** showing PC2 versus PC3 and highlighting the presence of CTCF.

Here we present an analysis of 208 CAP occupancy maps in the hepatocellular carcinoma cell line HepG2 performed as part of the ENCODE project, composed of 92 traditional ChIP–seq experiments with factor-specific antibodies and 116 CRISPR epitope tagging ChIP–seq (CETCh–seq) experiments^17,18^. Of all human CAPs, approximately 960 are expressed in HepG2 cells above a threshold RNA value of 1 FPKM (fragments per kilobase of transcript per million mapped reads), the lowest level at which we can routinely generate successful ChIP–seq and CETCh–seq results. This resource contains ChIP–seq and CETCh–seq maps for about 22% of these 960 CAPs, of which 171 are sequence-specific TFs and 37 are histone-binding or histone-modifying proteins, or other chromatin regulators or transcription cofactors (Fig. 1a, Supplementary Table 1). This large and unbiased sampling in one cell type allowed us to approach analysis from complementary directions, beginning with patterns of CAP occupancy and co-occupancy to find preferential associations with each other and with promoters, enhancers, or insulator functions, and in the other direction, working from genomic loci, sequence motifs, and epigenomic states to explain occupancy. These publicly available ENCODE occupancy data, together with the analyses and insights presented here, comprise a key resource for the scientific community.

- We analyse ChIP–seq and CETCh–seq maps for about 22% of TFs and other CAPs expressed in the human HepG2 cell line.
- We use clustering to classify major groups of CAPs, including those that are promoter- or enhancer-associated, or that are associated with both promoters and enhancers to a similar extent.
- Using this large amount of data, we demonstrate that DNA sequence motifs or ChIP–seq peak calls can distinguish between promoters and enhancers.
- We show that high-occupancy target (HOT) regions are driven by strong motifs for one or a few TFs and weaker, more degenerate motifs for many other CAPs.

## CAPs segregate regulatory element states

**Extended Data Fig. 1:**
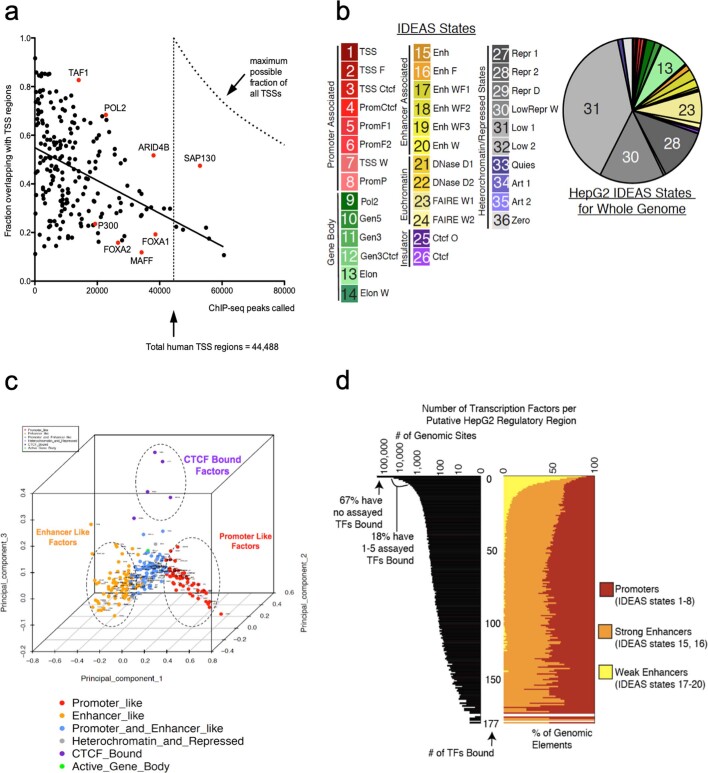
CAP associations with annotated TSSs and IDEAS regions. **a**, The 208 ChIP–seq and CETCh–seq experiments plotted by number of peaks called in each experiment (*x*-axis) against fraction of peaks overlapping with any of 44,488 TSSs in the human genome (peaks ±3 kb from TSS). Selected individual CAPs are labelled. Solid line is linear regression through all points; dotted lines represent number of total TSS regions and maximum possible fraction of TSSs. **b**, IDEAS segmentation of HepG2 cell genome. Left, colour key for all IDEAS states; right, pie chart indicating fraction of HepG2 genome associated with each state. **c**, Clustering of 208 CAPs on the basis of chromatin state recapitulating the assigned cluster, with PC1 (63.50%), PC2 (16.51%) and PC3 (6.48%) variances explained. **d**, Left, distribution of regulatory regions by number of associated CAPs; right, distribution of horizontally matched sites by IDEAS state.

As an initial analysis, we investigated how the binding of each of the 208 CAPs is distributed in the genome relative to known transcriptional promoters. We calculated the fraction of each of the called peaks of each CAP that was within 3 kilobases (±3 kb) of transcription start sites (TSSs), analysing only the TSSs of genes expressed (≥1 TPM (transcripts per kilobase million)) in HepG2 cells (Fig. 1b) and, separately, all annotated TSSs regardless of expression (Extended Data Fig. 1a). Individual CAPs exhibited variable proportions of promoter-associated peaks, independent of the number of peaks called in an experiment.

To further summarize the occupancy landscape, we merged all the called peaks from every experiment into non-overlapping 2-kb windows, limited to those windows in which two or more CAPs had a called peak, and performed principal component analysis (PCA) on these DNA segments, using the presence or absence of each CAP at each genomic segment. This analysis captured global patterns of ChIP–seq peaks, with principal component 1 (PC1) explaining about 28% of the variance and correlating strongly with the number of unique CAPs associated with a given genomic region (Fig. 1c). PC2 separates promoter-proximal from promoter-distal peaks, underscoring the relevance of promoters as a predictor of genomic state and CAP occupancy (Fig. 1d). Notably, the shape of this plot suggests that, as the number of CAPs associated at a locus increases, the promoter-proximal and promoter-distal regions lose separation along PC2. In addition, PC2 plotted against PC3 shows strong segregation based on occupancy of the factor CTCF (Fig. 1e), suggesting that discrete genomic demarcations are attributable to this factor, as expected given its insulator and loop-anchoring functions.

To assess the epigenomic context of each binding site, we used IDEAS (integrative and discriminative epigenome annotation system), a machine-learning method for biochemical mark-based genomic segmentation^19^. This IDEAS HepG2 epigenomic segmentation inferred 36 genomic states based on eight histone modifications, RNA polymerase ChIP–seq, CTCF ChIP–seq, and DNA accessibility data sets (DNase and formaldehyde-assisted isolation of regulatory elements (FAIRE)). Notably, IDEAS states for HepG2 cells were classified using mainly histone marks, augmented by only two chromatin-associated ChIP–seq maps included in our data set (CTCF and RNA polymerase). These segregate the anticipated major classes of correlations between epigenomic states in the IDEAS segmentation and CAP associations, such as enrichment of H3K4me3 at annotated promoters and H3K27ac at candidate active enhancers, as well as open chromatin status as assayed by DNA accessibility experiments, typical of TF-bound DNA. As expected, the resulting IDEAS states classified only a minority of the HepG2 genome as potential *cis*-regulatory elements (Extended Data Fig. 1b).

**Fig. 2:**
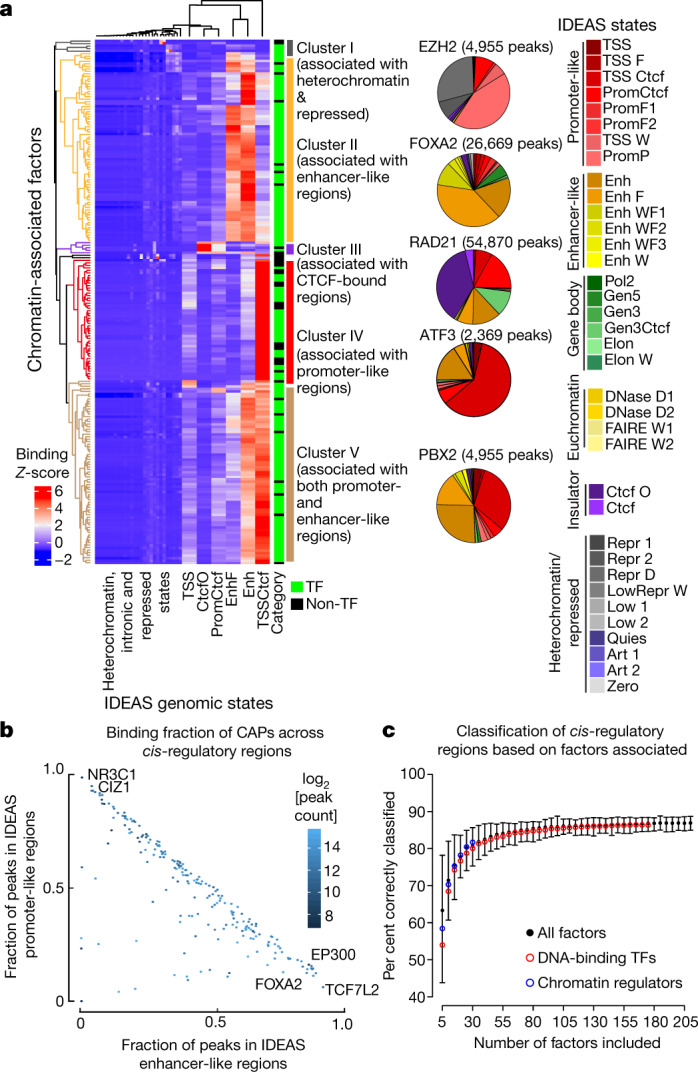
Landscape of factor binding to regulatory states. **a**, Unsupervised clustering of the 208 factors on the basis of binding enrichment at 36 IDEAS genome states and the 5 main clusters of factors, along with pie charts showing absolute binding fractions of an example of a factor from each cluster. **b**, Correlation plot showing the fraction of promoter (*y*-axis) or enhancer (*x*-axis) binding for all 208 factors, with points coloured by peak counts for each factor. **c**, Predictive ability of random forest classification of genomic regions as either enhancer or promoter on the basis of the number of factors used to train the algorithm; *n* = 100 iterations, lines from minimum to maximum with median indicated.

We calculated the relative IDEAS state enrichments of the peak calls for each CAP, and clustered the CAPs by these enrichments. The resulting matrix delineated several clear bins of genomic state associations, expanding and refining the previously noted preferential proximal versus distal genomic associations of CAPs^20^. Specifically, we found a subset of CAPs that are preferentially associated with promoters, another subset associated with candidate active enhancers, and a third group distributed across both proximal promoter regions and candidate active enhancers (Fig. 2a). We also found two smaller CAP-associated clusters: one associated with heterochromatin and repressed marks (including BMI1 and EZH2, both part of Polycomb repressive complexes), and one with CTCF regions (including CTCF and the known cohesin complex proteins RAD21 and SMC3; Fig. 2a, Supplementary Table 2). These categories contain members of different classes of CAPs, and point to distinct gene regulatory pathways. A PCA based on these IDEAS states also recapitulated these clusters (Extended Data Fig. 1c).

**Extended Data Fig. 2:**
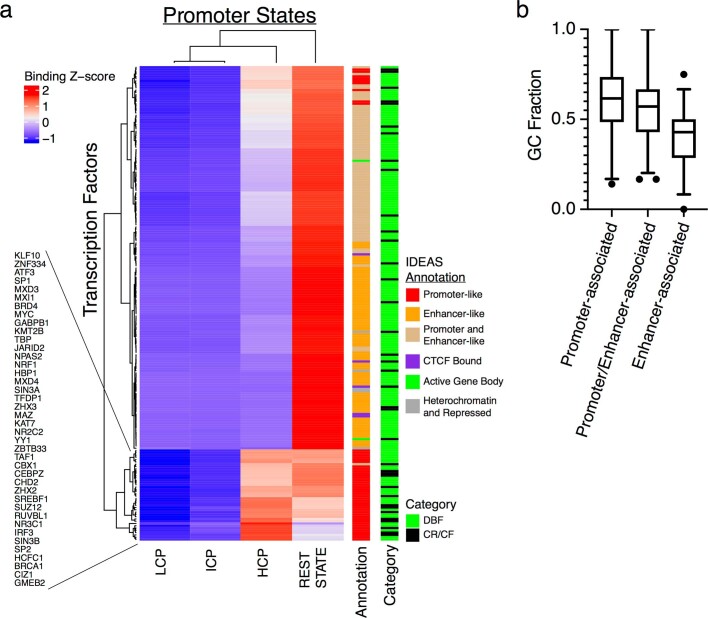
CAP associations with varying CpG and GC content. **a**, Heat map and clustering of CAPs on the basis of association with low, intermediate, and high CpG content promoters (LCP, ICP, and HCP, respectively). All regions outside promoters are denoted as rest state. Annotation from Fig. 2a is shown, as are categories of direct DNA-binding factors (DBFs) and chromatin regulators or cofactors (CR/CF). **b**, Box plot of GC content of motifs for CAPs associating with promoters (*n* = 26), with both enhancers and promoters (*n* = 45), or with enhancers (*n* = 55). Centre line, median; boxes, 25th–75th percentiles; whiskers, 5th–95th percentiles.

For roughly 40% of the CAPs assayed, most called peaks were in IDEAS promoter-like regions, while about 30% of CAPs were predominantly associated with IDEAS enhancer-like regions (Fig. 2b). Although these preferences are part of a continuous distribution, the unsupervised clustering using all IDEAS genomic states suggests that subsets of CAPS show strong localization preferences. We analysed whether the promoter-associated CAPs associated predominantly with CpG-island promoters by annotating promoter regions according to previous classifications for low, intermediate, and high CpG content^15,21^. The promoter-associated CAPs also cluster preferentially with promoters with high CpG content (Extended Data Fig. 2a, Supplementary Table 3). However, the GC content of motifs for CAPs in the promoter-associated cluster is not significantly different from that of CAPs associated with both promoters and enhancers, suggesting that motif GC content alone does not drive the clustering (Extended Data Fig. 2b).

The CAPs that associate with both promoters and enhancers do not have apparent bias in relation to the GC content of promoters. Previous publications have noted similarities between promoters and enhancers, ascribing enhancer activity to promoters, and transcription occurs directly at enhancers in the form of enhancer RNA (eRNA) and even as alternative promoters^22–24^. The subset of CAPs identified as associating with both promoters and enhancers may point to specific genomic loci or gene regulatory networks wherein the lines between promoters and enhancers are most blurred.

Because CAPs localize to specific genomic states, we were able to reproducibly train random forest models to predict the IDEAS state of a genomic region using binding information for only a small number of CAPs (Fig. 2c). The prediction method was successful when using a combination of TFs with chromatin regulators and other extended CAPs, but was also successful when trained only on direct DNA-binding TFs or only on non*-*TFs. Each approach required a subset of roughly any 30 CAPs to achieve approximately 80% accuracy.

## CAP distribution in regulatory elements

Although the 208 CAPs do not represent a complete catalogue of all expressed CAPs in HepG2 cells, we investigated how much of the regulation in this cell line is captured by this partial compendium. We used IDEAS to define a set of 370,570 putative HepG2 *cis*-regulatory elements classified as promoters, ‘strong’ enhancers, or ‘weak’ enhancers, with merging of similar features within 100 base pairs (bp), resulting in a broad size distribution from 200 bp to 12–16 kb. We then calculated how many CAPs were associated in each region (Extended Data Fig. 1d). On average there were seven CAPs associated at any putative regulatory region. Approximately 67% of the regions did not contain any called peaks; however, the vast majority of these (about 85.5%) were classified as ‘weak’ or ‘poised’ enhancers by the IDEAS segmentation. Conversely, elements classified as promoters or ‘strong’ enhancers by IDEAS were enriched for occupancy by higher numbers of CAPs (Extended Data Fig. 1d). Of the IDEAS-determined active promoter-like regions, 61% contained a called peak for at least one CAP in this data set, and of the strong enhancer-like regions, 75% contained at least one called peak. Because most promoters and strong IDEAS-modelled enhancers had one or more CAPs associated, and these elements had an average of 15 and 18 unique associated CAPs per region, respectively, these data capture a substantial overview of the CAP regulatory network in HepG2 cells.

## Motif analysis reveals CAP associations

**Fig. 3:**
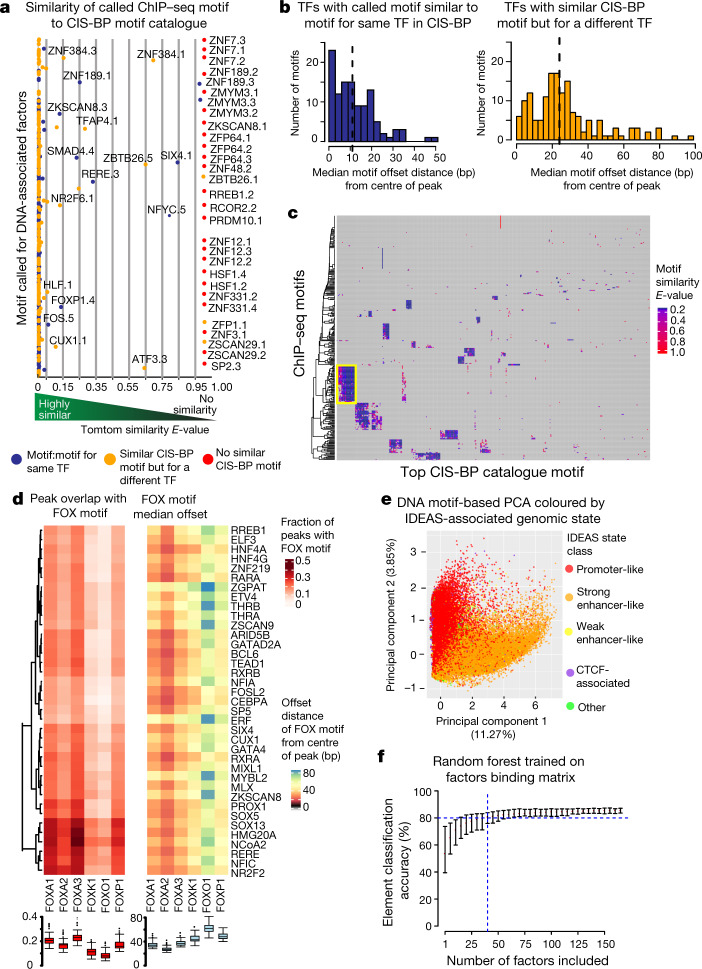
Motif identification and analysis. **a**, The 293 high-confidence motifs derived from analysis of the ChIP–seq data were quantitatively compared to all (human) motifs in the CIS-BP database and plotted according to similarity scores. Blue points represent motifs that matched the assayed factor, yellow points represent motifs that match a factor other than the one assayed, and red points represent motifs not similar to any in CIS-BP. **b**, Histograms showing the distance from the centre of the ChIP–seq peak for motifs that do (left) or do not (right) match the TF. **c**, Clustered heat map showing the similarity of all 293 significant motifs to 733 motifs from CIS-BP for the assayed factors. **d**, Further analysis of the cluster containing 37 factors that had FOX family motifs, showing the overlap of FOX TF binding in these peaks, as well as the median offset of the FOX motif from the centre of the ChIP–seq peaks. For box plots (bottom), *n* = 37 CAPs; boxes show middle quartiles, centre line shows median, whiskers show 1.5× interquartile range (IQR). **e**, PCA showing separation of motifs that fall in promoters versus those that fall in enhancers; *n* = 408,382 genomic elements. **f**, Prediction accuracy for calling whether an element is a promoter or enhancer on the basis of motifs that are present; *n* = 100 iterations, lines from minimum to maximum with median indicated.

**Extended Data Fig. 3:**
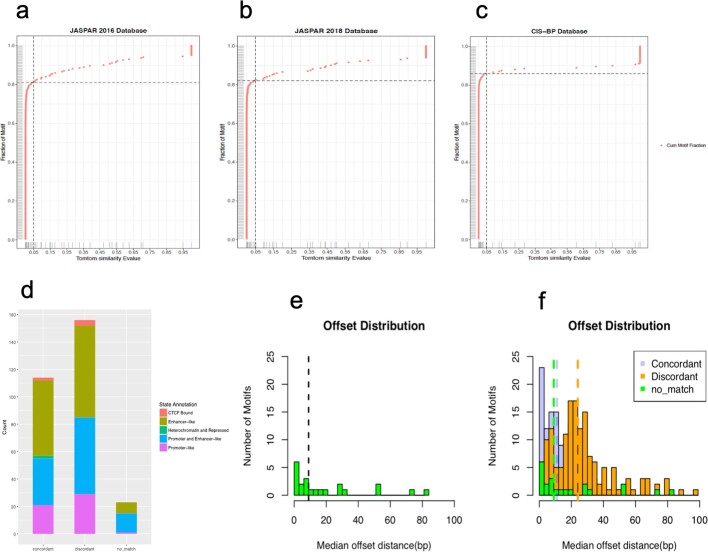
Motif analysis. **a**, Cumulative fraction of called motifs in our data compared to motifs in the JASPAR 2016 vertebrate database as scored by Tomtom similarity *E*-value. **b**, Cumulative fraction of called motifs in our data compared to motifs in the JASPAR 2018 vertebrate database as scored by Tomtom similarity *E*-value. **c**, Cumulative fraction of called motifs in our data compared to motifs in the CIS-BP (build 1.02) *Homo sapiens* database as scored by Tomtom similarity *E*-value. **d**, Distribution of TF motifs by concordance (matching expected TF), discordance (matching different TF), and no match in the CIS-BP database. Stacked bar plots are coloured by main TF groups from previous unsupervised clustering. **e**, Distribution of TF motifs highly dissimilar to all motifs in CIS-BP (*y*-axis) and their median offset distance from the centre of peaks (*x*-axis). **f**, Stacked distribution of highly dissimilar motifs (no match; green) with similar (concordant; blue) and motif called for secondary factor (discordant; orange) and their median offset distances from the peak centre (*x*-axis).

We assessed motif enrichment in peaks, and found many previously derived motifs for both direct and potentially indirect associations, as well as some potentially novel motifs. We derived a high-confidence set of 293 motifs called from 160 of the 171 putatively direct DNA-binding TFs in our data set^2^. We compared these motifs to the JASPAR databases^25,26^ and to the Catalog of Inferred Sequence Binding Preferences (CIS-BP) database^8^ to determine whether our de novo derived motifs matched previous findings from various in vivo and/or in vitro assays^27^. Overall, more than 80% of the 293 motifs had a similar motif in these databases (86% in CIS-BP build 1.02, 82% in JASPAR 2018, 81% in JASPAR 2016; Extended Data Fig. 3a–c). For 114 motifs derived from peaks for 89 unique TFs, the most similar motif in the database was annotated as the motif for the TF that was the target of the ChIP–seq or CETCh–seq assay, and we call these cases ‘concordant’ (Fig. 3a, Supplementary Table 4). There were 156 motifs derived from peak data for 99 TFs that were more similar to the database motif of a different TF, and we denote these as ‘discordant’. We also observed 23 motifs derived from peaks of 14 TFs that were highly dissimilar to any motifs in the databases and may be previously undescribed motifs. Most of these were from zinc finger TFs, a large class of factors that has been virtually unassayed by endogenous ChIP–seq.

We note that concordant calls were sometimes problematic, specifically when the motif in a database originated from a previous ChIP–seq experiment. In some cases, these motifs probably do not represent the specific sequence recognized by the TF assayed, but are spurious calls from associated TFs that replicate across multiple ChIP–seq experiments. For example, two motifs for ATF3 matched an ATF3 ChIP–seq motif in CIS-BP, which qualifies these motifs as concordant, but they more closely resemble an E-box motif. We overruled the automatic concordant call for this case, and manually changed it to discordant. For Supplementary Table 4, we curated each called motif to clarify results from the matching algorithm, and included a column with this information.

Among the 163 discordant motifs, motifs representing pioneer TFs such as FOXA1 were enriched, and we hypothesize that these motifs were called owing to their substantial co-occurrence with the assayed TFs. Previous studies have noted the enrichment in ChIP–seq data of sequences that do not appear to be binding motifs for assayed TFs, but rather are more similar to other TF motifs^28^. There are several potential explanations for why the ChIP–seq-derived motif would most closely match a motif previously annotated for another factor. Related TFs often recognize very similar sequence motifs; for example, the motif we derived for TEAD4 was very similar to the motif previously found for TEAD1^29^. There are also instances in which a CAP lacks a strong and specific DNA-binding domain and no motif would be expected unless the motif represents a frequent co-binding partner, a scenario we explore below with GATAD2A. A similar explanation involves a particular TF acting as an ‘anchor’ at a locus, and either through direct protein–protein interactions, or by inducing an open chromatin environment, behaving as a mechanism for localization of other proteins. A well-studied example of this highlighted in our data was the enrichment of the CTCF motif in RAD21 ChIP–seq data, as RAD21 lacks a DNA-binding domain but interacts with CTCF. It is difficult to determine confidently whether a discordant motif represents a key co-factor interaction or a commonly co-localized protein. When we called multiple, distinct, high-confidence motifs in a single ChIP–seq experiment, with one motif annotated in databases as the direct target of the assayed TF and another motif representing a different TF that we also assayed separately, the results of the secondary factor’s ChIP–seq experiment suggested that both TFs are likely to be associated at these loci, as both experiments yielded called peaks at these loci.

Supporting our hypothesis that, in the discordant cases, the motif of the secondary TF was not a site of direct binding for the primary CAP, examination of the precise location of the motifs within peaks showed a significant difference (Kolmogorov–Smirnov test, *P* = 2.481 × 10^−12^); the direct matching motifs of the assayed TFs were closer to the centres of called peaks and the discordant motifs for other TFs were more offset, providing evidence for co-occurrence at these locations (Fig. 3b). Direct interaction and co-recruitment between these pairs of TFs could explain these observations, and numerous examples of such combinatory and cooperative activities between TF pairs have been reported^30^. We found no significant trend for secondary TF motifs in any factor clusters we identified by IDEAS state preferences or other methods, suggesting that no biases were introduced by contributions from particular genomic loci (Extended Data Fig. 3d). We also analysed the peak locations of the 23 novel motifs found with the 14 factors that were highly dissimilar to any motifs in CIS-BP, and the majority showed enrichment at the centres of peaks (Extended Data Fig. 3e, f), supporting the notion that these are previously undescribed motifs for direct DNA binding by these TFs.

To better understand discordant TF motif calls, we constructed a similarity heat map using all 293 high-confidence motifs from our data and motifs for each assayed TF annotated in the CIS-BP database (*n* = 733; Fig. 3c). This analysis clustered TFs both by similarity of their direct binding motifs (such as all Forkhead factors) and by co-occurrence with other motifs. We thereby identified TFs that associate at genomic loci near particular motifs, such as CTCF. Most obvious was a set of 37 CAPs for which a Forkhead motif was called, indicating the high prevalence of this motif in HepG2 cells at active enhancers and promoters, and the key role of TFs such as FOXA1 and FOXA2 in the gene regulatory network in these cells. We examined these cases using our ChIP–seq data from six FOX TFs (FOXA1, FOXA2, FOXA3, FOXK1, FOXO1, and FOXP1), testing how often each of these FOX TFs yielded called peaks with a FOX motif that overlapped with a peak for any of these 37 other CAPs, and we found that most of the 37 contained a FOX peak with a FOX motif in about 20% of their peaks, with FOXA1 and FOXA3 motifs being the most common (Fig. 3d).

**Extended Data Fig. 4:**
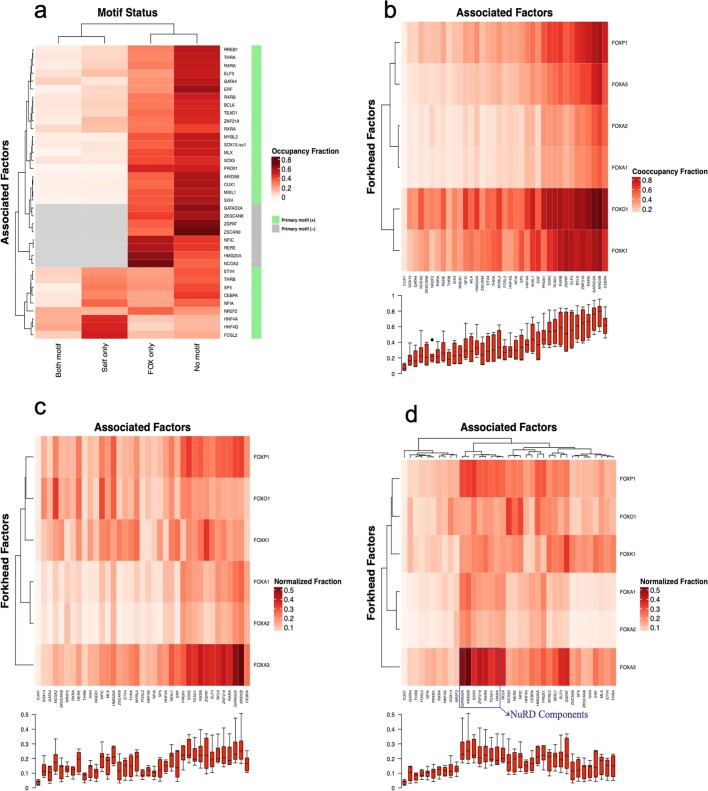
CAPs associated with FOX TFs and motifs. **a**, Thirty-seven non-FOX TFs with a called Forkhead motif, with heat map denoting fraction of called peaks with both a primary (matched to specific TF) motif and a FOX motif, with a primary motif but not a FOX motif, with a FOX motif but no primary motif, and with neither a primary nor a FOX motif. The eight TFs with grey boxes do not have a known primary motif. **b**, Peak overlaps between the 37 TFs and 6 FOX factors for which we obtained ChIP–seq data; box plots represent distribution of all FOX overlaps for each of the 37 factors. **c**, Same as **b**, but normalized for peak counts of each of the 37 factors. **d**, Same as **c**, but clustered vertically, revealing NuRD component clustering. Box plots are vertically matched, *n* = 6 overlap measurements; boxes, middle quartiles; centre line, median; whiskers, 1.5 × IQR.

We next examined the locations of the FOX motifs in the overlapping peaks and found that all were offset to varying degrees, always with a median distance of more than 20 bp from the centres of peaks (Fig. 3d). In addition, we examined all peaks called for each of the 37 CAPs and identified the fraction that contained a primary motif specific to the individual CAP (where known) along with a FOX motif, the fraction that contained only the primary motif, the fraction that contained only a FOX motif, and the fraction that contained neither motif (Extended Data Fig. 4a). For the 30 CAPs with a described motif, the majority of peaks did not contain a primary motif, a result that may indicate protein–protein interactions and/or looping events in these peaks. Furthermore, when we examined peak overlaps between these 37 TFs and the six FOX TFs, we observed varying associations and co-occupancy partners, including factor preferences for individual FOX TFs and a cluster of components of the nucleosome remodelling and histone deacetylase (NuRD) complex (Extended Data Fig. 4b–d).

Motif information alone was predictive of genomic segments, clearly showing segregation between IDEAS states in a PCA (Fig. 3e). A random forest algorithm trained only on motifs was able to predict IDEAS states almost as well as one trained on ChIP–seq peaks, achieving approximately 80% success with any roughly 40 motifs (Fig. 3f).

## Known and novel CAP associations

TFs and chromatin regulatory proteins can interact with and recruit other CAPs through direct and indirect physical associations. Although the activity of a few key CAPs may be very important for cell-state-specific expression, it is likely that combinatorial events are necessary to fine-tune expression^31^. We found both known and novel associations by examining occupancy overlaps and trends in a variety of analyses.

**Fig. 4:**
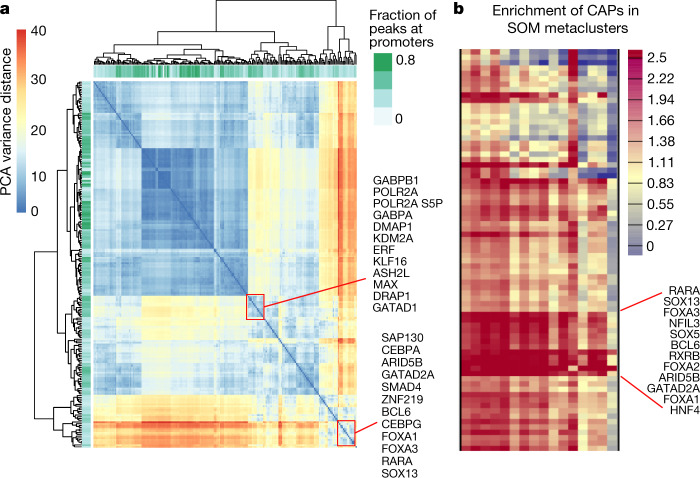
Co-localization of factors. **a**, Correlation matrix based on the cumulative principal component distances weighted by the proportion of variance explained by each component between all factors, derived from the PCA of all genomic loci with a peak containing at least two factors. **b**, SOM for a group of FOX TFs in HepG2 cells, with metaclusters showing major associations with specific factors.

To identify candidate co-occupancy events mediated by direct DNA binding or by indirect interactions, both of which produce peaks in ChIP–seq data, we performed several analyses. We used the PCA of the protein-bound genomic loci described above (in which genomic loci clustered according to the CAPs associated at each region; Fig. 1c–e), and generated a correlation matrix based on the cumulative PC distances (weighted by the proportion of variance explained by each component) between all CAPs. The resulting unsupervised clustering of respective pairwise distances highlighted punctate groups that represented both known and potentially novel complexes, including a group containing POL2 and TSS-associated chromatin-modifying enzymes and transcriptional cofactors, a group of cohesin complex members, a group of liver-specific factors (the tissue type from which HepG2 is derived), and a group containing the NuRD complex, among others (Fig. 4a).

**Extended Data Fig. 5:**
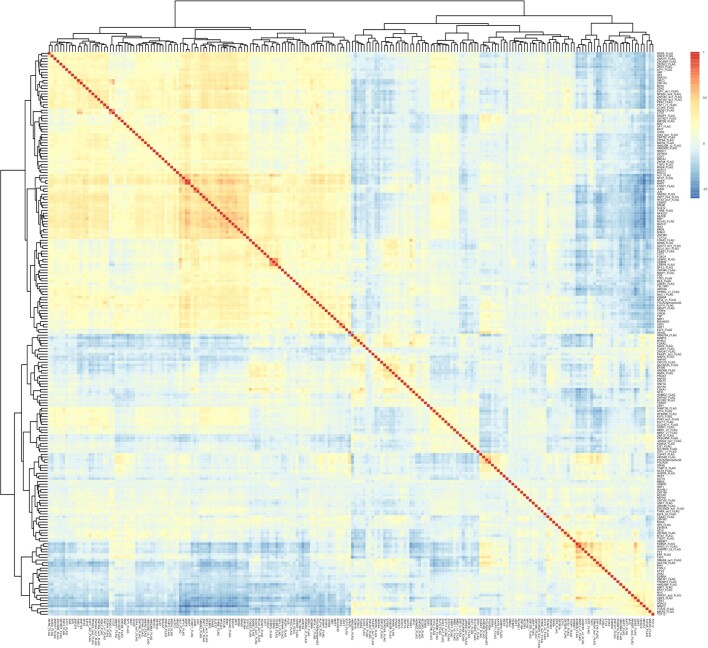
Read count correlations between CAPs. Read count correlations between all 208 assayed CAPs, mean centred and squared, with unsupervised clustering.

**Extended Data Fig. 6:**
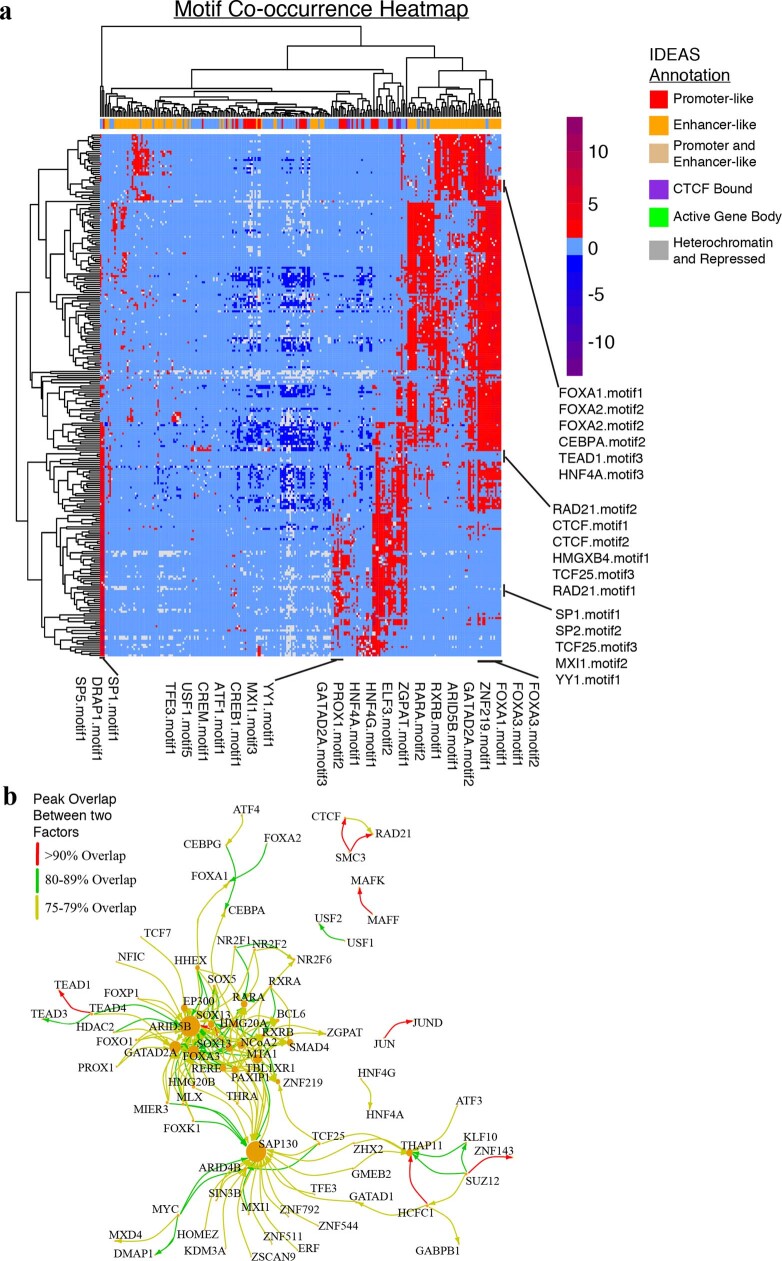
Motif and peak associations. **a**, Directional co-occurrence of motifs in ChIP–seq called peaks. **b**, Subset of network plot derived from peak overlaps between all factors, showing strong associations between a subset of factors.

To quantitatively analyse the overall data, we performed read count Spearman correlations between all 208 CAPs by calculating raw sequencing counts at every unique locus present in called peaks in any experiment (±50 bp from peak centre). The resulting correlation heat map also showed clusters of related CAPs as well as both known and potentially novel interactions (Extended Data Fig. 5, Supplementary Table 3). Network plots based on pairwise peak overlaps highlighted a number of known interactions, including CTCF–RAD21 and CEBPA–CEBPG networks, as well as CAPs that associate with a large number of other CAPs, usually chromatin regulatory proteins such as SAP130, GATAD2A, and ARID5B (Extended Data Fig. 6b). We examined the associations at the level of called motifs by finding the peaks in each experiment where a specific called motif was present, limiting the analysis to the 293 high-confidence motifs. Upon identification of the primary motif, we looked for associations between motifs 1–40 bp away (Extended Data Fig. 6a, Supplementary Table 3). This analysis revealed the TFs (and motifs) that were more likely to associate with the motif of any other particular TF. RAD21 was highly associated with CTCF motifs, as expected, and we also found several other known complexes as well as some novel associations. FOXA1 peaks with the canonical Forkhead motif were more likely to contain relatively few motifs for other factors, but many factors, such as HNF4A, HNF4G, and RXRB, were enriched for nearby FOXA1 motifs.

**Extended Data Fig. 7:**
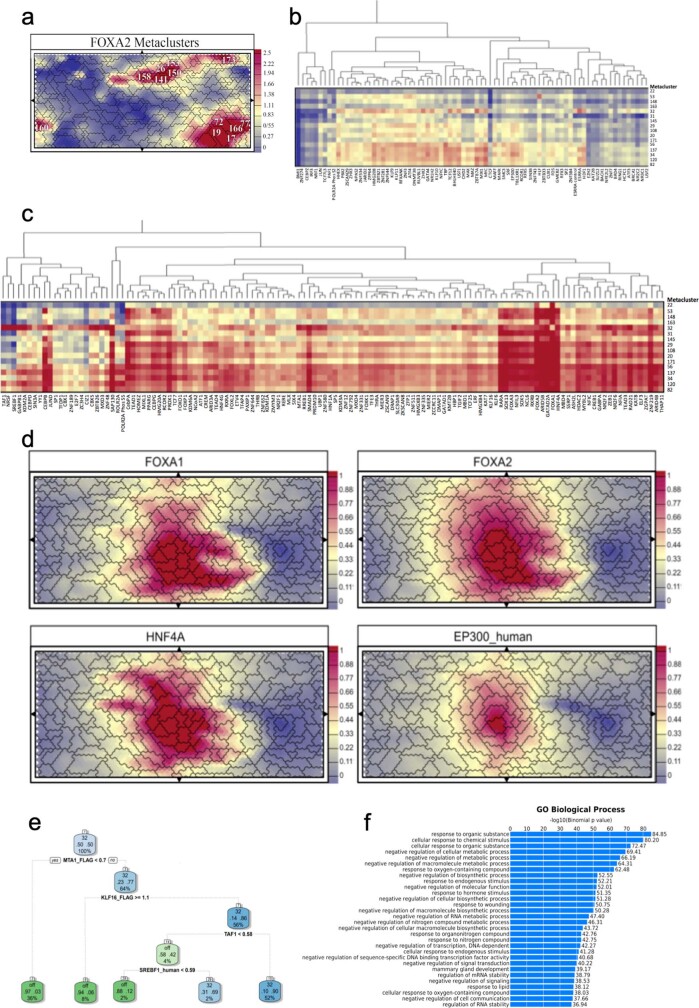
Self-organizing maps. **a**, SOM showing FOXA2 metaclusters. **b**, Example heat map showing CAP enrichment in 16 key SOM metaclusters. **c**, Example heat map showing CAP enrichment in 16 key SOM metaclusters. **d**, SOMs for FOXA1, FOXA2, HNF4A, and EP300. **e**, Example decision tree showing the presence or absence of CAPs for metacluster 32. **f**, GREAT analysis of metacluster 32-assigned genes that are likely to be regulated in this metacluster, and GO term analysis for these genes; *P* represents sample frequency probability.

To independently assess co-occupancy and provide an additional quantitative analysis, we trained a chromatin self-organizing map (SOM)^32^ using all 208 CAPs with the SOMatic package^33^. We found key metaclusters around the key HepG2 TFs FOXA1/2 and HNF4A, in association with CAPs that are important for liver development, nucleosome remodelling (NuRD complex), and cohesin subunits (Fig. 4b, Extended Data Fig. 7a–f, Supplementary Notes).

**Fig. 5:**
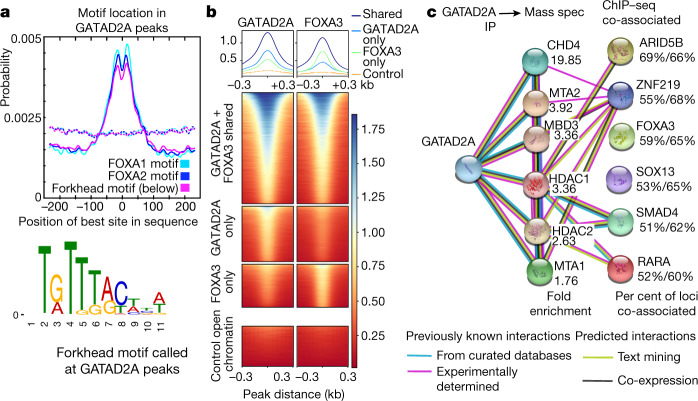
Analysis of GATAD2A co-localization. **a**, Presence of top motifs at GATA2DA-bound regions (top) and the top motif called at these peaks (bottom). **b**, Heat map showing signal intensity at shared and unique peaks for FOXA3 and GATAD2A. A set of random open chromatin regions is shown as a control. **c**, NuRD complex members and their identification through immunoprecipitation (IP)–mass spectrometry of GATAD2A immunoprecipitations, and through co-binding at GATAD2A-bound loci. Annotations from the String Database on protein interactions are shown as coloured lines connecting the proteins.

**Extended Data Fig. 8:**
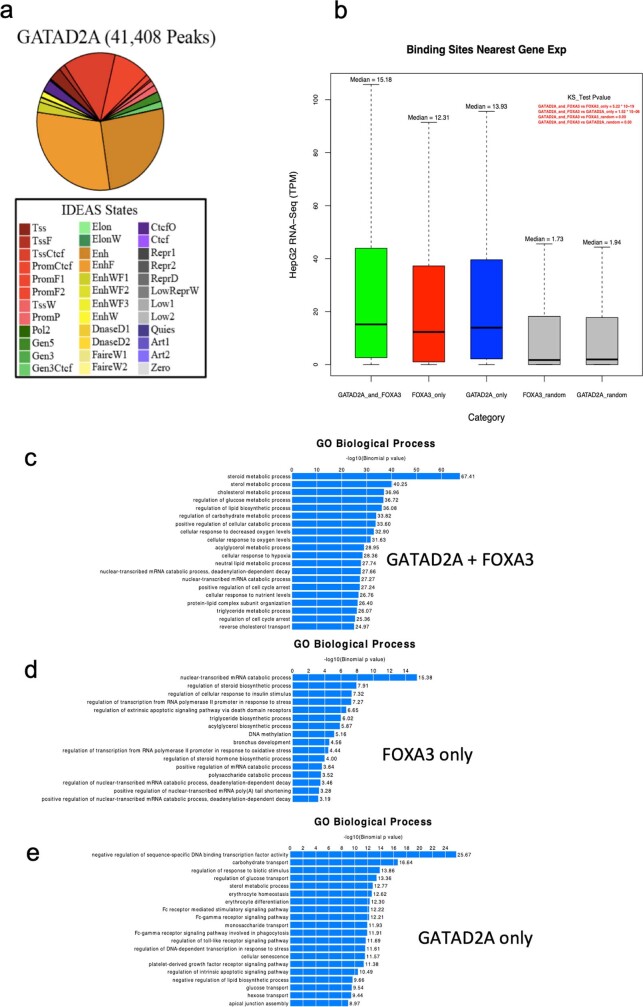
GATAD2A analyses. **a**, GATAD2A genome-wide ChIP–seq binding in HepG2 cells annotated by IDEAS state. **b**, Box plots showing expression level (RNA-seq TPM) of genes nearest sites with both GATAD2A and FOXA3 ChIP–seq peaks (green), genes nearest sites with FOXA3 peaks but no GATAD2A peaks (red), genes nearest sites with GATAD2A peaks but no FOXA3 peaks (blue), and GC-matched null regions for each CAP (grey). Boxes, middle quartiles; centre line, median; whiskers, 1.5 × IQR; *n* = 27,440 binding sites (GATAD2A + FOXA3), *n* = 10,658 binding sites (FOXA3 only), *n* = 13,706 binding sites (GATAD2A only), *n* = 37,073 binding sites (FOXA3 null matched), *n* = 40,441 binding sites (GATAD2A null matched). **c**, GO enrichments for genes with both GATAD2A and FOXA3 peaks. **d**, GO enrichments for genes with FOXA3 peaks but no GATAD2A peaks. **e**, GO enrichments for genes with GATAD2A peaks but no FOXA3 peaks. GO *P* value represents sample frequency probability.

The indirect motif, co-occupancy, and SOM analyses identified novel CAPs associated with GATAD2A, a core component of the NuRD complex. In GATAD2A CETCh–seq experiments, 53% of the GATAD2A peaks in HepG2 cells were annotated as active enhancers (Extended Data Fig. 8a), which was unexpected given the association of the NuRD complex with transcriptional repression and enhancer decommissioning^34–36^. GATAD2A has a very degenerate DNA-binding domain and is not predicted to bind DNA independently, and indeed the called GATAD2A motif matched FOXA3 (Fig. 5a). To assess co-localization in an additional, quantitative manner, we examined signal intensity^37^ at shared and unique sites for GATAD2A and FOXA3 (Fig. 5b). Many of the unique sites showed signal above background, indicating a limitation of the conservative peak calls we used and adding support for extensive co-localization for these factors.

In our co-association analysis in HepG2 cells, we identified six CAPs that co-occurred with GATAD2A in discrete genomic regions (Fig. 5c). We analysed GATAD2A–FLAG protein immunoprecipitation by mass spectrometry and found that multiple components of the NuRD complex also co-immunoprecipitated with GATAD2A (Supplementary Table 5). Of the GATAD2A-associated CAPs, ZNF219^38^, SMAD4^39^, and RARA^40^ have previously been associated with the NuRD complex (Fig. 5c). We additionally identified ARID5B, SOX13, and FOXA3 (see above) as proteins that were associated with the known NuRD group, specifically at active enhancers where Forkhead binding sites were enriched (Fig. 5b, c). The classic NuRD complex has been suggested to function at enhancer regions associated with tissue-specific gene regulation^41^, and our data confirm that the core NuRD component GATAD2A is recruited into these regions. Note that NuRD binding at these open and presumably active regions is thought to function through a NuRD complex that contains MBD3 and not MBD2, and our GATAD2A–FLAG immunoprecipitation–mass spectrometry data confirmed this, as MBD3 peptides but not MBD2 peptides immunoprecipitated with GATAD2A^42^ (Supplementary Table 5).

We examined the expression of the genes nearest to peaks with both GATAD2A and FOXA3 association, as well as those with GATAD2A or FOXA3 binding but not both. All of these sites were near genes that were expressed at significantly higher levels than genes near random GC-matched sites (Extended Data Fig. 8b). Moreover, sites with both GATAD2A and FOXA3 peaks were near genes with significantly higher expression than those nearest sites with only GATAD2A or FOXA3 (Extended Data Fig. 8b). The genes nearest the GATAD2A–FOXA3 co-associated sites were enriched for liver biology gene ontology (GO) terms, including cholesterol metabolic processes and regulation of lipids, whereas FOXA3 sites without GATAD2A were near genes with additional liver biology GO terms, such as regulation of insulin and triglyceride biosynthesis, and GATAD2A sites without FOXA3 were enriched for negative regulation of sequence-specific DNA binding TFs (Extended Data Fig. 8c–e). Additional analyses indicated that there were strong associations between CAPs and important liver biology genes (Supplementary Notes, Supplementary Fig. 1).

## CAPS in highly occupied regions

**Fig. 6:**
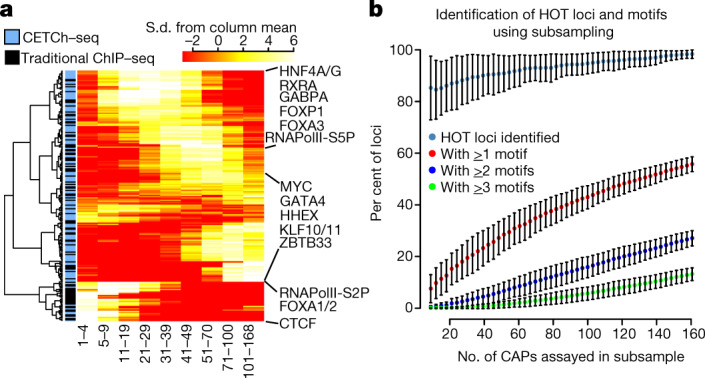
Association and motif trends in high CAP co-localization. **a**, CAP enrichment at loci with increasing number of factors bound. **b**, Subsampling plot showing the frequency of identification of motifs in HOT regions using increasing number of factors in permutations. Points represent median percentage of loci with one or more motifs (red), two or more motifs (dark blue), or three or more motifs (green) for CAPs bound at those regions; *n* = 100 iterations, lines from minimum to maximum.

We examined how many factors were bound at putative HepG2 *cis*-regulatory elements by merging all peaks from all 208 CAP experiments, with a maximum merged size of 2 kb. This analysis yielded a total of 282,105 genomic sites with at least one associated CAP, with a maximum of 168 CAPs at one site. We investigated whether certain CAPs were more likely to co-occupy genomic loci with a high number of other CAPs, by performing hierarchical clustering of the degree of co-association for each CAP; this resulted in three distinct clusters (Fig. 6a). The first was a cluster of 33 proteins, including previously described key pioneer factors such as FOXA1 and FOXA2^43^, which exhibit a low degree of co-occupancy with other CAPs at a relatively high proportion of their binding sites. The second cluster, comprised of 32 CAPs, displays frequent association at higher co-occupancy regions and is composed of CAPs already known to be recruited by, or to interact with, a large number of other CAPs, such as MYC and DNMT3B^44,45^. The third cluster contains the remaining CAPs, which exhibit an intermediate degree of co-occupancy, including key HepG2 TFs such as HNF4A and FOXA3.

**Extended Data Fig. 9:**
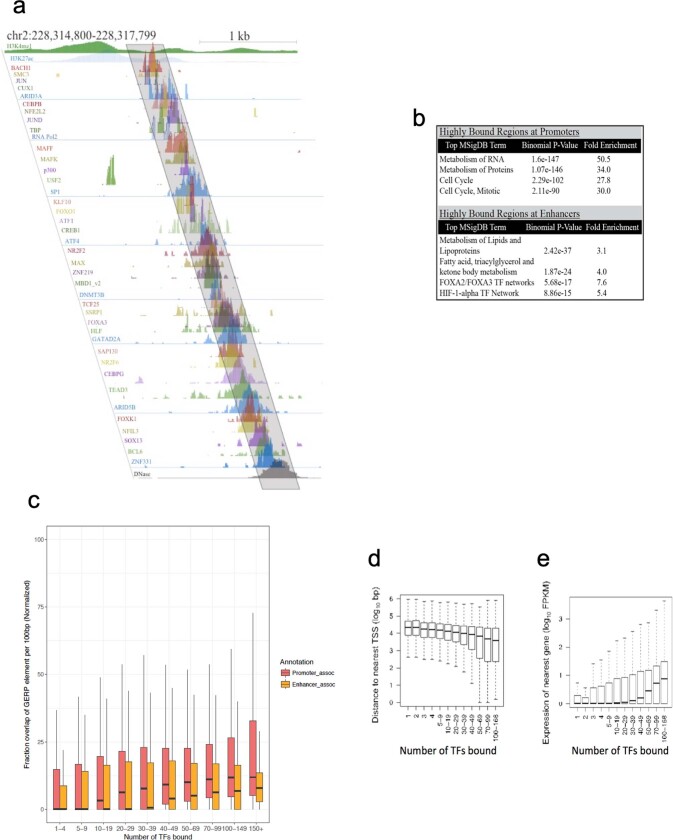
Extensive co-associations between CAPs. **a**, Example of genomic site with many associated CAPs. Each track shows aligned ChIP–seq reads, and is slightly offset to better show peaks for each experiment. **b**, Enrichment of biological pathways at HOT regions near enhancers or promoters; *P* represents sample frequency probability. **c**, Increasing numbers of CAPs bound at genomic sites correlate with increased evolutionary constraint as measured by GERP, showing incremental fraction overlap of highly constrained elements with CAP-associated sites for both promoter regions (red) and enhancer regions (orange). Boxes, quartiles; centre line, median; whiskers, 1.5 × IQR. **d**, Increasing numbers of CAPs bound at genomic sites (<2 kb in size) are associated with decreasing distance to nearest TSS; boxes, middle two quartiles; centre line, median; whiskers, 1.5 × IQR. **e**, Increasing numbers of CAPs bound at genomic sites (<2 kb in size) are associated with increasing expression of nearest gene; boxes, middle two quartiles; centre line, median; whiskers, 1.5 × IQR. **d**, **e**, Left to right: *n*(1) = 124,074, *n*(2) = 59,407, *n*(3) = 19,661, *n*(4) = 12,433, *n*(5–9) = 23,517, *n*(10–19) = 14,757, *n*(20–29) = 7,077, *n*(30–39) = 4,703, *n*(40–49) = 3,542, *n*(50–69) = 5,061, *n*(70–99) = 4,655, *n*(>100) = 3,219, total *n* = 282,105.

As previously described^46–48^, many regions in the genome are occupied by large numbers of CAPs in ChIP–seq assays (example shown in Extended Data Fig. 9a). There are several possible explanations for these HOT regions^49^. Some researchers have filtered all or the majority of these regions from analyses under the assumption that they are artefacts^50,51^. It is also possible that they are the result of stochastic shuffling of direct binding of many CAPs in a population of cells; when assayed across the millions of cells used for an individual ChIP–seq experiment, this could result in apparent co-localization of peaks for many CAPs that do not actually co-occupy at the same time in the same cell. The mechanisms that underlie this phenomenon might include indiscriminant recruitment driven by key CAPs or some unknown property of these regions of open chromatin, or by densely packed DNA sequence motifs. Another possible explanation is that three-dimensional genomic interactions, including enhancer looping and/or protein complexes, lead to ChIP–seq cross-linking of CAPs in close proximity.

We define HOT regions in these data (*n* = 5,676) as those sites with more than 70 CAPs (about one-third of all assayed CAPs) within a 2-kb region. Intersecting HOT regions with IDEAS segmentations revealed that more than 92% of HOT regions map to candidate promoter or strong enhancer-like states (42.25% and 49.88%, respectively). We determined using GREAT (genomic regions enrichment of annotations tool) analysis that promoter-localized HOT regions are associated with housekeeping genes and that distal HOT regions are near genes associated with liver-specific pathways (Extended Data Fig. 9b). In addition, the number of CAPs correlates with sequence conservation of the putative regulatory element and with the level of expression of the nearest gene (Extended Data Fig. 9c–e). While previous researchers have noted apparent general ChIP bias in favour of highly expressed genomic regions^51^, we performed ChIP in untagged cells with an antibody raised against the epitope tag used in CETCh–seq experiments, normalizing for this background in peak calling, and the HOT regions continued to be strongly enriched (data not shown).

**Extended Data Fig. 10:**
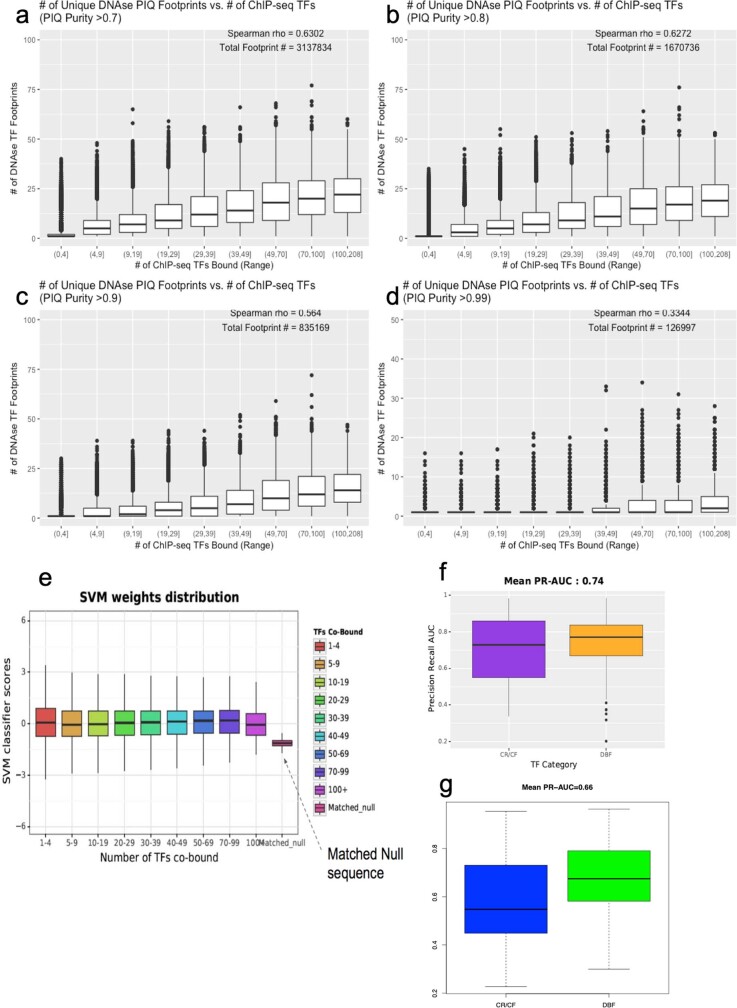
PIQ and SVM analyses in CAP co-associated regions. **a**, Number of unique DNase PIQ footprints (*y*-axis) plotted by sites with varying numbers of associated CAPs (*x*-axis), for PIQ threshold >0.7. **b**, Number of unique DNase PIQ footprints (*y*-axis) plotted by sites with varying numbers of associated CAPs (*x*-axis), for PIQ threshold >0.8. **c**, Number of unique DNase PIQ footprints (*y*-axis) plotted by sites with varying numbers of associated CAPs (*x*-axis), for PIQ threshold >0.9. **d**, Number of unique DNase PIQ footprints (*y*-axis) plotted by sites with varying numbers of associated CAPs (*x*-axis), for PIQ threshold >0.99. **a**–**d**, Boxes, middle two quartiles; whiskers 1.5 × IQR; centre line, median; *n*(0–4) = 216,496, *n*(4–9) = 23,540, *n*(9–19) = 14,859, *n*(29–39) = 4,947, *n*(39–49) = 3,735, *n*(49–70) = 5,517, *n*(70–100) = 3,995, *n*(100–208) = 1,681. **e**, Distribution of SVM classifier scores (*y*-axis) for sites with varying numbers of associated CAPs (*x*-axis). The scores remain relatively constant across sites and are significantly higher than the scores of classifier values in matched null sites. Boxes, middle two quartiles; whiskers 1.5 × IQR; centre line, median; *n*(1–4) = 1,814,475 bins, *n*(5–9) = 643,997 bins, *n*(10–19) = 646,453 bins, *n*(20–29) = 330,795 bins, *n*(30–39) = 194,981 bins, *n*(40–49) = 118,622 bins, *n*(50–69) = 131,167 bins, *n*(70–99) = 57,819 bins, *n*(100+) = 3,545 bins, *n*(matched null) = 9,597,800 bins. **f**, SVM PR-AUC scores for non-TFs (chromatin regulators and cofactors; CR/CF) and for TFs at motif-level mean PR-AUC (0.74). **g**, SVM PR-AUC scores for non-TFs (chromatin regulators and cofactors) and for TFs at motif-level mean PR-AUC (0.66). **f**, **g**, Boxes, middle two quartiles; whiskers 1.5 × IQR; centre line, median; *n*(CR/CF) = 37, *n*(DBF) = 171.

We computationally examined the general DNA motif structure of the HOT sites using two analyses. We first used a subsampling test to test whether motif information was gained as the numbers of CAPs assayed increased. We ran permutations of 12–162 CAPs and determined how often we could identify a HOT region as being bound by more than 33% of the CAPs in the subsample (Fig. 6b). More than 80% of the HOT loci were identified with only ten factors, and the curve approached 100% as the number of CAPs increased. We then investigated how often the motif for any associated CAP was found; fewer than 20% of sites had even a single motif identified with 40 or fewer CAPs. However, once more than 130 factors were included, over half the sites contained one or more identifiable motifs. While this analysis required only motif presence, we also found evidence of direct DNA–protein interactions using protein interaction quantification (PIQ)^52^—a computational tool that uses DNase-seq experiments and user-supplied motif sequences to identify direct TF binding sites. Using TF footprints identified in ENCODE HepG2 DNaseI hypersensitivity data by PIQ, we observed that the number of TF footprints was significantly positively correlated with the number of CAPs that had called peaks in a locus (Extended Data Fig. 10a–d). This observation was true at multiple PIQ purity (positive predictive value) thresholds and also when using TF footprints called in the same data set from JASPAR motifs. This is consistent with TF motif-driven architecture being a major characteristic of HOT regions. To determine whether CAP occupancy at highly bound regions is driven by specific DNA motifs, we trained a support vector machine (SVM) on the sequences of ‘HOT-motif’ sites, a set of peaks with 50 or more co-localized motifs derived from the HOT sites (*n* = 2,040). We tested the predictive ability of the SVM as the number of TFs increased and found that predictions remained constant, rather than declining, further strengthening the notion that these sites are not artefacts (Extended Data Fig. 10e). The average precision recall area under curve (PR-AUC) scores for the SVM were about 0.74 for motif-level predictions and about 0.66 for peak-level predictions. These scores were substantially higher than expected, given the random sample of a positive set of 5,000 sites tested against 50,000 GC-matched null sequences as the negative set (Extended Data Fig. 10f). We also found, using the *k*-mers generated by the SVM, that there are 1–5 TFs at each site with very high motif scores, and about 25–50 TFs with degenerate or weaker motifs (Extended Data Fig. 10g); this was true for both HOT-motif sites and the broader HOT sites.

**Extended Data Fig. 11:**
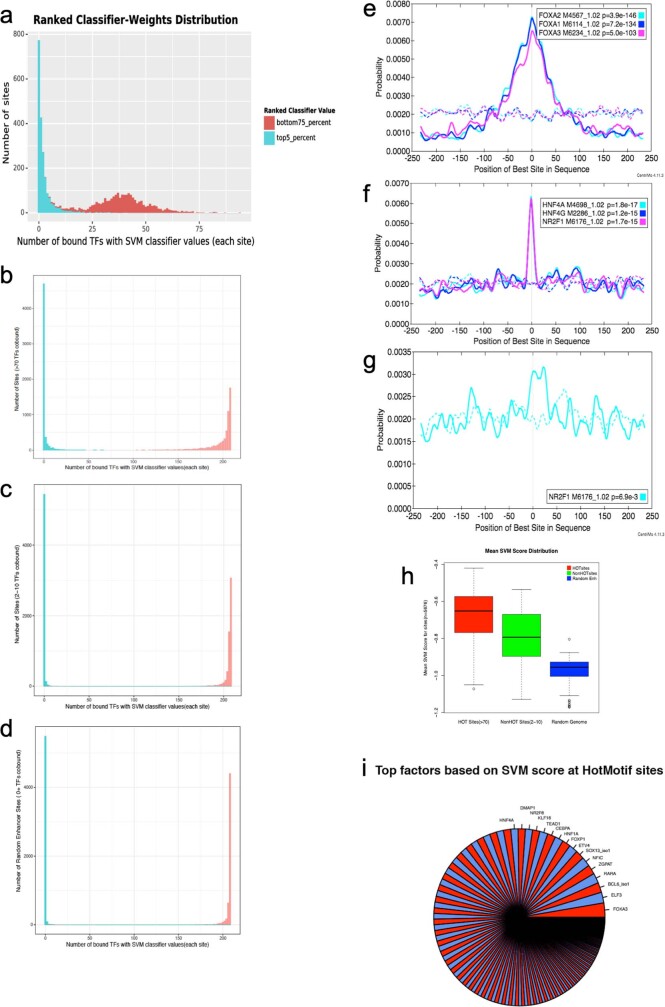
SVM and motif analyses in HOT sites. **a**, Number of sites (*y*-axis) by measured number of TFs (*x*-axis) with classifier values in the top 5% of all classifier values (blue) or with classifier values in the bottom 75% of all classifier values (red) in highly bound regions, based on SVM scores of factor peaks associated with highly bound regions. **b**, Number of sites (*y*-axis) by measured number of TFs (*x*-axis) with classifier values in the top 5% of all classifier values (blue) or with classifier values in the bottom 75% of all classifier values (red), in HOT sites with >70 associated TFs. **c**, Number of sites (*y*-axis) by measured number of TFs (*x*-axis) with classifier values in the top 5% of all classifier values (blue) or with classifier values in the bottom 75% of all classifier values (red), in sites with 2–10 associated TFs. **d**, Number of sites (*y*-axis) by measured number of TFs (*x*-axis) with classifier values in the top 5% of all classifier values (blue) or with classifier values in the bottom 75% of all classifier values (red), in a random set of enhancers with any number of associated TFs (0+). **e**, Degree of motif enrichment in highly bound regions for all HepG2-expressed TFs with available motifs (*n* = 365) for top three motifs enriched in highly bound sites with 50+ CAPs (highest *P* = 3.9 × 10^−146^). **f**, Degree of motif enrichment in highly bound regions for all HepG2-expressed TFs with available motifs (*n* = 365) for top three motifs in enhancers with 2–10 CAPs (highest *P* = 1.8 × 10^−17^). **g**, Degree of motif enrichment in highly bound regions for all HepG2-expressed TFs with available motifs (*n* = 365) for top motif in random genome enhancers with 0+ CAPs (highest *P* = 6.9 × 10^−3^). **h**, Distribution of all SVM scores (*y*-axis) for HOT sites with >70 associated CAPs (red), for sites with 2–10 associated CAPs (green), and for random enhancer sites with 0+ CAPs (blue). **i**, Pie chart showing fraction of HOT sites in which each TF has the highest SVM classifier value, indicating the strongest motif present.

We investigated whether this observation was unique to HOT regions (*n* = 5,676) when compared to an equal number of enhancer regions (as defined by IDEAS segmentation) with only 2–10 associated CAPs, or to a null set of random enhancer elements with any number (0–208) of associated CAPs. Sites with 2–10 CAPs had substantially smaller numbers of both high-affinity and low-affinity TF motifs, and the random enhancers were essentially devoid of strong motifs (Extended Data Fig. 11a–g). The distribution of SVM scores in HOT sites was significantly higher than that of the SVM scores of sites with 2–10 associated CAPs (Kolmogorov–Smirnov test, *P* = 5.966 × 10^−11^), and both were significantly higher than that of the null set of random enhancer elements (Kolmogorov–Smirnov test, *P* < 2.2 × 10^−16^ for each), indicating that the information imparted by the DNA sequence of HOT sites exceeds that of other *cis*-regulatory elements (Extended Data Fig. 11h). Moreover, in HOT sites, the strongest-affinity TF at any individual peak varied across sites, indicating that many different CAPs are involved in regulation at these sites. Important liver TFs, such as FOXA3, HNF1A, and CEBPA, had the strongest putative motif affinity at many of these sites (Extended Data Fig. 11i). This supports the notion that HOT sites are driven by a few strong and specific TF–DNA interactions and non-specific recruitment of other factors, probably through both protein complexes and binding to degenerate motifs, and possibly linking together multiple distal genomic regions through CAP interactions. Thus, it is essential to generate complete CAP maps to determine the full complement of CAPs associated with each locus, which would not occur by analysis of functional motifs alone.

## Discussion

This study introduces a data resource of occupancy maps for human transcription factors, transcriptional cofactors, histone-binding or histone-modifying proteins, and other chromatin regulators that illustrates the strengths of building towards a complete catalogue of CAP interactions in an individual cell type. At this intermediate stage of completeness, the aggregated data enabled us to identify known complexes and associations, and to identify putative novel associations. We also gained insights into gene regulatory principles, clearly showing the segregation of categories of CAPs associated with particular genomic states, including promoters and enhancers, and uncovering DNA sequence motifs at the majority of HOT regions that would have been impossible with fewer CAPs assayed.

The large number of CAPs assayed provided the capacity to identify and study regions of the genome associated with very high numbers of CAPs, compared with expectations from detailed work on specific enhancer complexes such as the interferon enhanceosome^53^. Multiple lines of evidence argue that, as a group, the regions at which high numbers of CAPs were detected are neither biological noise associated with general open chromatin nor ChIP–seq or CETCh–seq artefacts. HOT regions have been previously described as being depleted of TF motifs, but we suggest that this was likely to be because earlier analyses lacked a large enough sampling of key TFs with strong ‘anchoring’ motifs. We propose a model in which HOT regions are nucleated by anchoring DNA motifs and their cognate TFs. They would form a core, with which many other CAPs associate by presumed protein–protein interactions, protein–RNA interactions, and relatively weak DNA interactions at poorer sequence–motif matches. Extensive apparent co-occupancy at domains possessing few or no anchor motifs can potentially be explained when the ChIP assay captures, through assumed protein–protein fixation, non-adjacent DNA regions that associate with each other by looping interactions.

It is important to appreciate that the standard ChIP assay is performed on populations of large numbers of cells. Patterns of computational co-occupancy cannot discriminate between the simultaneous association of many CAPs in a single large molecular complex and diversified smaller complexes that are distributed at any given time across the cell population, with each containing a smaller number of secondary associations, which sum to give massive computational co-occupancy. We can, however, state that at individual known transcriptional enhancers with more than 70 CAPs, the ChIP signal for identified anchor factors was significantly higher in magnitude than at enhancers with fewer CAPs.

The results thus far argue that a fully comprehensive catalogue of all CAPs will help us to distinguish among these possibilities, which are not mutually exclusive. Completeness should also contribute to the identification of additional novel motifs, and, in the cases of indirect motifs found for TFs with known direct motifs, allow more accurate motif calling. In addition, a complete catalogue of CAPs in a single cell type will support the imputation of critical contacts in CAP networks for three-dimensional assembly of genomic enhancer–promoter organization that is not possible from a few individual CAP binding maps, as demonstrated by our findings regarding the NuRD complex. The ENCODE Project continues to produce additional occupancy maps and to expand cellular contexts for these assays. We anticipate more large-scale analyses such as this, and hope that the perspectives gained from these will inform more targeted research endeavours and generate meaningful hypotheses.

## Methods

### ChIP–seq and CETCh–seq

All protocols for ChIP–seq and CETCh–seq have been previously published and are available at the ENCODE web portal (https://www.encodeproject.org/documents/). In brief, HepG2 cells were obtained from ATCC (HB-8065), confirmed by morphological observation, and tested for mycoplasma (ThermoFisher C7028). Pools of cells were grown separately to represent replicate experiments. Crosslinking of cells was performed with 1% formaldehyde for 10 min at room temperature and the chromatin was sheared using a Bioruptor Twin instrument (Diagenode). Antibody characterization standards are published on the ENCODE web portal and consist of a primary validation (western blot or immunoprecipitation–western blot) and a secondary validation (immunoprecipitation followed by mass spectrometry) for traditional antibody ChIP–seq. With CETCh–seq experiments, a molecular validation (PCR or Sanger sequencing confirmation of edited genes) in addition to one of the immunological validations (western blot, immunoprecipitation–western blot, or immunoprecipitation–mass spectrometry) is required for release. Raw fastq data were downloaded from the publicly available ENCODE Data Coordination Center, and aligned to the human reference genome (hg19) using the BWA-0.7.12 (Burrows Wheeler Aligner) alignment algorithm^54^. Post-alignment filtering steps were carried out using samtools-1.3^55^ with MAPQ threshold of 30, and duplicate removal was performed using picard-tools-1.88 (http://broadinstitute.github.io/picard/). After filtering, each CAP’s genome-wide binding sites (peak enrichment) were computed using phantompeakqualtools, implementing the SPP algorithm^56,57^, with replicate consistency and peak ranking determined by irreproducible discovery rate (IDR) using the IDR-2.0.2 tool^56^ to generate narrow peaks passing IDR cutoff 0.02 (soft-idr-threshold). ENCODE blacklisted regions (wgEncodeDacMapabilityConsensusExcludable.bed.gz, downloadable from the UCSC genome browser at https://genome.ucsc.edu/) were filtered out. In addition, we note that plasmids used to generate edited cells with epitope-tagged CAPs have been deposited to Addgene, the non-profit plasmid repository, and are available for researchers to tag particular CAPs in other cell lines of interest. We also note that the GC content of DNA has been reported as a source of bias in ChIP–seq data, leading to over-representation of TFBSs and false positive peak calls, which could confound subsequent analyses^58,59^. To address this concern, we performed ChIP–seq experiments in unedited cell lines using the FLAG antibody (Sigma F1804) that we use in CETCh–seq, and used these libraries as background for peak calling. In these experiments, the only variable is the edited cell line used as foreground, and most biases should be accounted for.

### De novo sequence motif analysis

To identify enriched sequence motifs in the binding sites of CAPs, de novo sequence motif and motif enrichment analysis were performed using the MEME-ChIP^60^ suite and the pipeline was built as previously described^61^, on 500-bp regions centred on peak summits based on the hg19 reference genome fasta. The top five motifs per data set were reported from the top 500 peaks based on signal value, using 2× random/null sequence with matched size, GC content and repeat fraction as a background. Central motif enrichment analysis was performed using Centrimo^62^, to infer the most centrally enriched motifs with de novo motifs generated from the pipeline against the 2× null sequence background.

### Comparative motif analysis

De novo motifs generated from CAPs were filtered for high-confidence motifs, including only those that were highly significant and strongly enriched in binding sites, based on MEME *E* < 1 × 10^−5^, Centrimo *E* < 1 × 10^−10^ and Centrimo binwidth <150. High confidence motifs were then compared, and quantified for similarity against the previously derived or known motifs available in the CIS-BP build 1.02 and JASPAR 2016/2018 databases^8,25,26^ using the Tomtom quantification tool^63^. Tomtom *E*-values <0.05 represent highly similar motifs, and >0.05 represent motifs with increasing magnitude of dissimilarity, or more distantly related motifs.

### Gene expression

RNA-seq quantification data for 56 cell lines and 37 tissues were retrieved from the Human Protein Atlas (version 17, downloadable from https://www.proteinatlas.org/)^64^, and used to identify 57 genes that were highly and specifically expressed in liver as compared to all other cell and tissue types, and also found in HepG2 cells with at least 10 TPM. On average, these 57 liver-specific genes were 151.21 times more highly expressed than in any other cell type.

### IDEAS segmentation

IDEAS segmentation for six cell-types (HepG2, GM12878, H1hESC, HUVEC, HeLaS3, and K562) were collected from the Penn State Genome Browser (http://main.genome-browser.bx.psu.edu/). All promoter-like and enhancer-like regions identified in at least one of five other cell lines were merged using pybedtools^65,66^ and these regions were filtered from the HepG2 segmentation. Significant enrichment of CAPs in the *cis*-regulatory regions was evaluated using Fisher’s exact test (adjusted *P* < 0.001, BH FDR corrected) against random or null sequences with matched length, GC content and repeat fraction using null sequence python script from Kmer-SVM^67^. Heat maps were generated using the heatmap.2 function from R gplots package (https://cran.r-project.org/web/packages/gplots/).

### GREAT analysis

*Cis*-regulatory associated highly CAP bound sites were binned into promoter-associated and enhancer-associated sites using IDEAS segmentation. To assess the biological function and relevance of these highly occupied sites, GREAT^68^ analysis was performed to predict the function of these *cis*-regulatory regions (http://bejerano.stanford.edu/great/public/html/) by associating the genomic regions to genes from various ontologies such as GO molecular function, MSigDB and BioCyc pathway. The parameters used for GREAT analysis were Basal+extension (constitutive 5.0 kb upstream and 1.0 kb downstream, up to 50.0 kb max extension) for all enhancer-associated sites, and Basal+extension (constitutive 5.0 kb upstream and 1.0 kb downstream, up to 5.0 kb max extension) for all promoter-associated regions with whole-genome background. MSigDB pathway^69,70^ was noted for genomic region enrichment analysis.

### GERP analysis

Genomic evolutionary rate profiling (GERP) was performed to assess whether highly bound *cis*-regulatory sites, categorized into promoter or enhancer-associated, correlate with increased evolutionary constraints. A highly constrained elements bed file containing high-confidence regions (significant *P*) generated from per base GERP scores was retrieved from the Sidow laboratory at Stanford (http://mendel.stanford.edu/SidowLab/downloads/gerp/). The fraction of overlapping bases for each bin of the ‘CAP bound category’ (low to high) with highly constrained elements was computed using bedtools-2.26.0^66^ and pandas-0.20.3, python2.7, further normalized by the fraction of ‘highly constrained elements’ overlapping per 100-bp region of CAP bound categories. In addition, the Kolmogorov–Smirnov test was performed to evaluate statistically significant differences in distribution between the highly bound (20+ CAP bound) and not highly bound regions (1–19 CAP bound sites) for both promoter- and enhancer-associated sites.

### Co-binding analysis

Pairwise overlap of binding sites between each of the 208 CAPs was performed with 50 bp up- and downstream from the summit of peaks using python-based pybedtools^65,66^. All other computations, and the pairwise peak overlap percentage for each CAP to build the pairwise matrix, were performed using pandas-0.20.3, python2.7 (Python Software Foundation) to construct network plots, using R igraph, implementing the Fruchterman Reingold algorithm. The interconnection between CAP shared binding sites for 208 CAPs was built with a minimum threshold of 75% or more overlap between any two CAPs. The sizes of vertices and nodes in the graph are representative of the number of connections each CAP has with its connected partner, while edges represent the degree of overlap between CAPs.

Co-binding was characterized by merging IDR-passing narrow peak files from 208 CAPs with the ‘merge’ function from the bedtools software package^71^. A minimum of 1 bp overlap was required and resultant peaks greater than 2 kb (~1%) were filtered from downstream analysis. Hierarchical clustering, using the Euclidean distance metric and Ward clustering method, of CAPs based on degree of co-binding was performed in R with the ‘heatmap.2’ function of the gplots package.

### LS-GKM SVM analysis

At peak level, LS-GKM support vector machines (SVMs)^72^ were trained on a random sample of up to 5,000 narrow peaks (using all peaks for those with fewer) as a positive set against 10× random/null sequence with matched size, GC-content and repeat fraction as a negative set. At motif level, LS-GKM support vector machines (SVMs)^72^ were trained on a sample of 5,000 random motif sites found by FIMO (MEME-suite), extending ±15 bp, for all TFs (*n* = 171), as a positive set against the 10× random-null sequence with GC content and repeat fraction matched sequence as a negative set.

Null genomic sequences matched to observed binding events were obtained using the ‘nullseq_generate.py’ function available with the LS-GKM package. The fold number of sequences (−*x*) was set to ten and the random seed (−*r*) was set to 1. SVMs were trained using the ‘gkmtrain’ function with a *k*-mer length (−*l*) of 11, kernel function (−*t*) of 4, regularization parameter (−*c*) of 1, number of informative columns (−*k*) of 7, and maximum number of mismatches (−*d*) of 3. Precision-recall areas under the curve (PR-AUC) were calculated by obtaining the tenfold cross-validation results from ‘gkmtrain’ (after setting the –*x* flag to 10), and inputting the results into the ‘pr.curve’ function of the PRROC R package, resulting in mean PR-AUC of 0.66 at the peak level, and 0.74 at the motif level. Classifier values for all bound sequences were obtained using the ‘gkmpredict’ function, and HOT sites (*n* = 5,676) were scored with each CAP to assess their putative binding affinity at HOT regions, and percentile ranked to obtain the top 5% and bottom 75% *k*-mer compared to enhancers with 2–10 associated TFs (*n* = 5,676) and to random enhancers with any number of associated factors (0+) (*n* = 5,676).

### Random forest and PCA analysis

PCA was performed on a CAP binding matrix composed of the presence or absence of motif in merged peaks as a binary matrix of loci, and implementing the python-based ML library scikit-learn Sklearn (0.19.0)^73^. Plots for motif-based analyses were generated using the R package ggplot2^74^ and complex Heatmap^75^. A random forest classifier was trained on merged CAP binding matrices at both motif and peak level to predict *cis*-regulatory elements (promoter or enhancer, by IDEAS annotation) using the R package ranger^76^, a faster implementation of random forest in R, and also tested using Sklearn 0.19.0. The median OOB (out-of-bag) error estimate was computed for 100 instances of randomly sampled (*n* = 1,000) loci iterations, to compute the element classification and misclassification accuracy using confusion matrix.

### Immunoprecipitation with mass spectrometry

Whole-cell lysates of FLAG-tagged or unedited HepG2 cells (~20 million) were immunoprecipitated using a primary antibody raised against FLAG or the CAP, respectively. The immunoprecipitation fraction was loaded on a 12% TGX gel and separated with the Mini-PROTEAN Tetra Cell System (Bio-Rad). The whole lane was excised and sent to the University of Alabama at Birmingham Cancer Center Mass Spectrometry/Proteomics Shared Facility. The sample was analysed on a LTQ XL Linear Ion Trap Mass Spectrometer by liquid chromatography electrospray ionization with tandem mass spectrometry (LC–ESI–MS/MS). Peptides were identified using SEQUEST tandem mass spectral analysis with probability based matching at *P* < 0.05. SEQUEST results were reported with ProteinProphet protXML Viewer (TPP v4.4 JETSTREAM) and filtered for a minimum probability of 0.9. For ENCODE antibody characterization standards, all protein hits that met these criteria were reported, including common contaminants. Fold enrichment for each protein reported was determined using a custom script based on the FC-B score calculation^77^. Following ENCODE antibody characterization guidelines, the CAP must be in the top 20 enriched proteins identified by immunoprecipitation–MS, and the top CAP overall for release. For GATAD2A co-associated TFs, the peptides with minimum 0.9 probability were present in smaller quantities than those of GATAD2A.

### TF footprints analysis

To identify TF footprints for comparison to ChIP–seq binding sites, we used PIQ^52^. ENCODE HepG2 DNase-seq raw FASTQs (paired-end 36 bp) of roughly equivalent size (accession numbers: ENCFF002EQ-G, -H, -I, -J, -M, -N, -O, -P) were downloaded from the ENCODE portal and processed using ENCODE DNase-seq standard pipeline (available at https://github.com/kundajelab/atac_dnase_pipelines) with flags: -species hg19 -nth 32 -memory 250G -dnase_seq -auto_detect_adapter -nreads 15000000 -ENCODE3. Processed BAM files were merged and used as input for PIQ TF footprinting using each TF’s top motif position weight matrix (PWM). Next, identified TF footprints from every TF that met a specified PIQ purity (positive predictive value) were intersected with all identified ChIP–seq binding sites using BEDtools to correlate the number of unique TF footprints with the number of ChIP–seq factors identified at a given ChIP–seq binding site.

### SOM analysis

The SOM was trained with the SOMatic package^33^ using the previous chromatin analysis partitioning strategy^32^ with modifications as described below. We calculated the RPKM of each data set’s first replicate over each of the 951,022 genomic segments to build a training matrix. We used each data set’s second replicate to build a separate scoring matrix. The training matrix was used to train five trial self-organizing maps with a toroid topology with size 40 × 60 units using 10 million time steps (~10 epochs) and selected the best, based on fitting error using the scoring matrix, for further analysis, and segments were assigned to their closest units based on the scoring matrix.

To properly fit the data, SOM units with similar profiles across experiments were grouped into metaclusters using SOMatic. In brief, metaclustering was performed using *k*-means clustering of the unit profiles to determine centroids for groups of units. Metaclusters were built around these centroids so that all of the units in a cluster remained connected. SOMatic’s metaclustering function attempts all metacluster numbers within a range given and scores them on the basis of Akaike information criterion (AIC)^78^. The penalty term for this score is calculated using a parameter called the dimensionality, which is the number of independent dimensions in the data, which in this case are the individual cell subtypes. To estimate this number, we used a 60% cut on a hierarchical clustering done on the SOM unit vectors. For this work, the dimensionality was calculated to be 6. For metaclustering, all *k* between 50 and 250, with 64 trials, were tested and metacluster number 196 had the lowest AIC score and was chosen for further analysis.

To generate decision trees for these metaclusters, each of the segments in the training matrix was labelled with its final metacluster. For each metacluster, if the metacluster is of size *n*, *n* segments of other clusters were chosen randomly, and this set of positive and negative examples was split, using 80% of the examples for training and 20% for scoring. The training data were fed through an R script using the rpart and rattle packages to create, score, prune, and re-score a tree for each metacluster. This entire process was repeated for 100 trials with only the tree with the highest accuracy drawn.

### Reporting summary

Further information on research design is available in the Nature Research Reporting Summary linked to this paper.

## Online content

Any methods, additional references, Nature Research reporting summaries, source data, extended data, supplementary information, acknowledgements, peer review information; details of author contributions and competing interests; and statements of data and code availability are available at 10.1038/s41586-020-2023-4.

## Supplementary information

Supplementary Information This file contains Supplementary Notes (A. Additional introductory material; B. Liver-specific TFs and genes reveal the cis- and trans-networks of HepG2; and C. SOM analysis), Supplementary Fig. 1, and additional references.

Reporting Summary

Supplementary Tables This file contains Supplementary Tables 1-6.

## Data Availability

Data sets generated from this study are available at the ENCODE portal or at the Gene Expression Omnibus under accession number GSE104247. CETCh–seq reagents are available at https://www.addgene.org/crispr/tagging/.

## Code availability

All code is available at https://github.com/chhetribsurya/PartridgeChhetri_etal.
