# Supplementary material for: Occupancy maps of 208 chromatin-associated proteins in one human cell type
Source: Nature. 2020 Jul 29;583(7818):720–8. doi: 10.1038/s41586-020-2023-4 (PMC7398277; doi:10.1038/s41586-020-2023-4)
Supplement: Supplementary file 1 — This file contains Supplementary Notes (A. Additional introductory material; B. Liver-specific TFs and genes reveal the cis- and trans-networks of HepG2; and C. SOM analysis), Supplementary Fig. 1, and additional references. [file 41586_2020_2023_MOESM1_ESM.pdf]

nature

https://doi.org/10.1038/s41586-020-2023-4

Supplementary information

Occupancy maps of 208 chromatin-associated proteins in one human cell type

In the format provided by the authors and unedited

E. Christopher Partridge, Surya B. Chhetri, Jeremy W. Prokop, Ryne C. Ramaker, Camden S. Jansen, Say-Tar Goh, Mark Mackiewicz, Kimberly M. Newberry, Laurel A. Brandsmeier, Sarah K. Meadows, C. Luke Messer, Andrew A. Hardigan, Candice J. Coppola, Emma C. Dean, Shan Jiang, Daniel Savic, Ali Mortazavi, Barbara J. Wold, Richard M. Myers ✉ & Eric M. Mendenhall ✉

Supplementary Notes

Additional introductory material

According to the most recent census and review of putative TFs, including manual curation of DNA-binding domains in protein sequences and experimental observations of DNA binding, there are 1,639 known or likely TFs in the human genome[2]. However, other tallies[1,7], and broader definitions of proteins that associate with DNA, including transcriptional cofactors such as RNA polymerase-associated proteins, histone-binding regulators, and chromatin modifying enzymes, suggest there are likely >1,800 and possibly as many as 2,500 such proteins encoded in the human reference genome assembly; we refer to these collectively as chromatin-associated proteins (CAPs) to distinguish this broad group of proteins from the stricter definition of direct DNA-binding TFs. A typical TF binds preferentially to a short DNA sequence motif, and, *in vivo,* some TFs also exhibit additional chromosomal occupancy mediated by their interactions with other CAPs[8-10], although the extent and biological significance of most secondary associations are not well understood[79]. CAPs play vital roles in orchestrating cell type- and cell state-specific gene regulation, including the temporal coordination of gene expression in developmental processes, environmental responses, and disease states[3-6,11-13].

Identifying genomic regions with which a TF is physically associated, commonly referred to as TF binding sites (TFBSs), is an important step toward understanding its biological roles. The most common genome-wide assay for identifying TFBSs is chromatin immunoprecipitation followed by high-throughput sequencing (ChIP-seq)[14-16]. In addition to highlighting potentially active regulatory DNA elements by direct measurement, ChIP-seq data can define specific DNA sequence motifs that can be used, often in conjunction with expression data and chromatin accessibility maps, to infer likely binding events in other cellular contexts without performing direct assays. Elegant methods have been

developed for identifying motifs[62,80-82], including ones that consider the plasticity of individual bases within and adjacent to a motif[83-86], account for structural details in relation to TF co-occurrence[87-89], or incorporate directly measured and inferred motifs[8]. Subsets of motifs can be specific to different cell types or environmental contexts, and can depend on the chromatin state and presence of cofactors for accessibility[90,91], and the presence of a motif sequence alone is often not predictive of a binding event[92-94]. While motifs identified by enrichment in ChIP-seq are often representative of direct binding, this is not always the case, as co-occurrence of other TFs could lead to the enrichment of their motifs. Further, the ChIP-seq method identifies both protein:DNA and, indirectly, protein:protein interactions, such that indirect and even long-distance interactions (e.g. looping of distal elements) can be captured as ChIP-seq enrichments.

A long-term goal is comprehensive mapping of all CAPs in all cell types, but a compelling and more immediate aspiration is to create a deep map of all CAPs expressed in a single cell type. The resulting consolidation of hundreds of genome-wide maps for a single cellular context promises insights into CAP networks that are otherwise not possible. Such comprehensive data will also provide the necessary backdrop for understanding large-scale functional element assays, and should improve the ability to infer TFBSs in other cell types that are less amenable to direct measurements.

Previous analyses of sets of numerous CAPs have been performed[95-99]. However, the larger studies to date have assayed occupancy by transfected CAPs, often expressed ectopically and at non-physiological levels, in contrast to this study, in which we performed assays on endogenous proteins expressed at physiological levels. This work in the HepG2 hepatocellular carcinoma cell line is part of the Encyclopedia of DNA Elements (ENCODE) Consortium effort toward achieving “factor completeness” (e.g., the mapping of all expressed CAPs’ binding locations) in a subset of commonly used human cell lines. We present here an analysis of 208 CAP occupancy maps in HepG2, composed of 92 traditional ChIP-seq experiments with factor-specific antibodies and 116 CETCh-

seq (CRISPR epitope tagging ChIP-seq) experiments. We developed the CETCh-seq method to address the dearth of ChIP-competent antibodies for many factors, and this method has been shown to be a robust, powerful assay[17,18]. Its strength is that the endogenous CAPs are tagged with a universal epitope that is recognized by a single well-characterized ChIP antibody, and that the tagged factors are expressed at physiological levels to avoid ectopic ChIP peaks that can be caused by conventional transgene overexpression[100,101]. As more CETCh-seq experiments are performed, the growing database is used to identify any antibody-specific artifacts attributable to cross-reactivity. This is part of the ENCODE Consortium quality control process for ChIP-seq, CETCh-seq, and related assays[56], which includes immune reagent validation and characterization by assays such as Western blots, and validation of tagged cell lines by confirmation of genomic DNA sequence. Additionally, the hundreds of ChIP experiments performed have led to tuning and optimization of protocols to alleviate technical biases[102,103]. Results of validation experiments for CAPs assayed here are available on the ENCODE web portal at www.encodeproject.org.

Of the >1,800 total human CAPs, approximately 960 are expressed in HepG2 cells above a threshold RNA value of 1 FPKM (Fragments Per Kilobase of transcript per Million mapped reads), the lowest level at which we can routinely generate successful ChIP-seq and CETCh-seq results. The resource we present here contains ChIP-seq and CETCh-seq maps for ~22% of these 960 CAPs, of which 171 are sequence-specific TFs and 37 are histone-binding/-modifying proteins or other chromatin regulators or transcription cofactors (Fig. 1a and Supplementary Table 1). This large and unbiased sampling in one cell type allowed us to approach analysis from complementary directions, beginning with patterns of CAP occupancy and co-occupancy to find preferential associations with each other and with promoters, enhancers, or insulator functions, and in the other direction, working from genomic loci, sequence motifs, and epigenomic state to explain occupancy.

All ChIP-seq/CETCh-seq data are available through the ENCODE web portal (www.encodeproject.org), or at Gene Expression Omnibus. We identified each CAP's genome-wide binding sites by using the SPP algorithm[57], with replicate consistency and peak ranking determined by Irreproducible Discovery Rate (IDR)[104]. This publicly available ENCODE occupancy data, together with analyses and insights presented here, comprise a key resource for the scientific community.

Liver-specific TFs and genes reveal the cis- and trans-networks of HepG2

Identifying transcription networks is important for understanding how genes specify a cell type. Our current understanding is that TFs, including key cell-type specifying factors, interact with other factors via combinatorial cross-regulation to drive gene expression in a cell-specific manner. To identify HepG2-specific cis-regulatory elements, we used IDEAS segmentation to identify all promoter-like and enhancer-like regions in at least one of five other cell lines (GM12878, H1hESC, HUVEC, HeLa-S3, and K562), and filtered these regions from the HepG2 segmentation. In the resulting set of 59,115 putative HepG2-specific cis-regulatory regions, we found significant enrichment (Fisher's exact test, adjusted p-value <0.001, BH FDR corrected) of distinctive CAPs at HepG2-specific enhancer loci, including known important liver TFs such as HNF4A, HNF4G, CEBPA, and FOXA1, along with additional CAPs not previously associated with liver cell identity such as TEAD1, RXRB, and NFIL3 (Supplementary Fig. 1a).

Because HepG2 is a cancer cell line derived from liver tissue, we focused on liver-specific genes, filtering for genes that are highly and specifically expressed in liver and also expressed in HepG2 at levels of at least 10 TPM. This identified a total of 57 key liver/HepG2 specific genes. We then examined the peak calls of all 208 CAPs close to promoter regions of the 57 liver specific genes (+/- 2 kb from TSSs), finding between 13 and 148 CAPs associated with promoters of each of these genes. Pioneer TFs (capable of binding closed chromatin and

usually involved in recruiting other factors[105,106]) such as FOXA1, FOXA2, and CEBPA, as well as key chromatin regulators such as EP300, associate with most of the liver-specific genes (Supplementary Fig. 1b). Of note, the promoters of the very highly expressed liver genes *ALB*, *APOA2*, *AHSG*, *FGA*, and *F2* (also known as thrombin) have very high apparent factor occupancy/association: 65, 148, 124, 114, and 130 CAPs, respectively (Supplementary Fig. 1c). We examined CAP occupancy at the promoters of all genes as well as of those genes expressed at 10 TPM or higher in HepG2, and compared these to CAP occupancy at the 57 liver-specific genes (Supplementary Fig. 1d-f, Supplementary Table 6). In each analysis, increasing factor number correlates positively with increasing RNA level. We note that some prior studies suggested that TF occupancy at highly expressed loci is a technical artifact of ChIP-seq[51], but, as described below in the section on HOT sites, several lines of evidence argue that these signals represent true biology. The 57 liver-specific genes have significantly higher expression (rank percentile t-test; p-value < 0.0001) when compared to other genes matched by number of CAPs, indicating a trend toward higher expression associated not only with a higher number of associated CAPs but with specific factor identities. The CAPs that are associated with higher than expected expression based on the number of CAPs associated at their promoters include unsurprising examples such as PAF1 and RNA polymerase II subunit A (Ser2 phosphorylated), marks of active transcription, as well as ATF4 and HSF1 (Supplementary Fig. 1g). However, we note that there are still many CAPs that have not yet been assayed by ChIP-seq, and this could explain some of the deviation from expected expression. An additional caveat is that each experiment is normalized separately, thus limiting comparison of relative activity levels of individual CAPs.

SOM analysis

For an independent assessment of co-occupancy and as an additional quantitative analysis, we trained a chromatin self-organizing map (SOM)[32] using all 208 CAPs with the SOMatic package[33]. This analysis generated 196 distinct

clusters of SOM units, with each such “meta-cluster” sharing similar profiles, and corresponding decision trees that trace the supervised learning path used to determine the unique features of each metacluster profile (Fig. 4c, Extended Data Fig. 7). Focusing on the key HepG2 TFs FOXA1/2 and HNF4A, we found that 18 distinct metaclusters accounted for nearly half of the peaks for these 3 TFs (43% for FOXA1, 43% for FOXA2, and 49% for HNF4A). CAPs important for liver development, nucleosome remodeling, and the cohesin complex show high co-binding signal in these key 18 metaclusters.

Looking closer at the CAPs that distinguish these 18 key clusters, we found that five of these metaclusters (numbered as 32, 34, 56, 120, and 137) show strong signal from CEBPB, SAP130, and RAD21. In particular, metacluster 32 had a collection of unique features related to the NuRD complex and liver processes. A decision tree trained on regions in this cluster highlighted the presence of TAF1 and MTA1 (part of the NuRD complex) and the absence of a high signal of KLF16 (a known TF displacer) as sufficient to predict association with MBD1, HBP1, and HDAC2 (a sub-unit of the NuRD complex) with ~91% accuracy. GREAT (Genomic Regions Enrichment of Annotations Tool[68]) analysis of these regions revealed a related set of negative regulation and response GO (Gene Ontology) terms, which provides further evidence that the NuRD complex is involved in tissue specific gene regulation.

Supplementary Figure 1

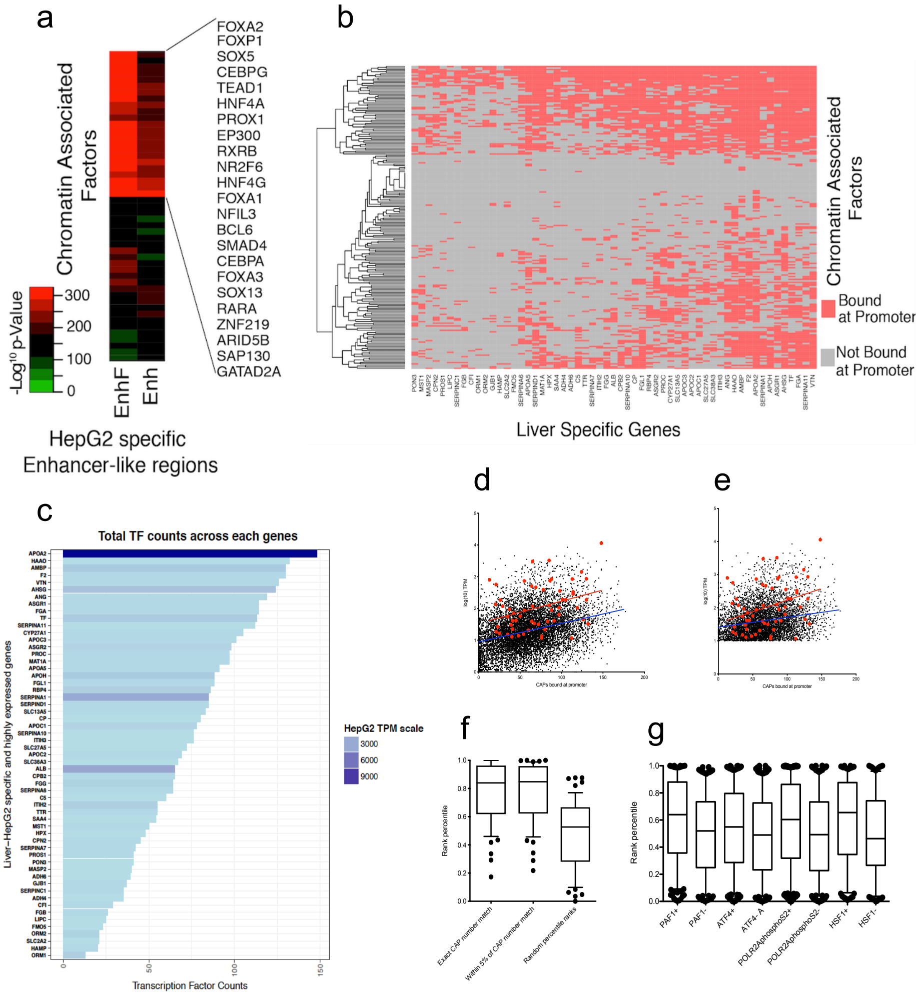

Supplementary Figure Legend

Supplementary Fig. 1: CAPs associated with genes important in liver biology

a, Enrichment of CAPs at regions of the genome we classified as putative HepG2-specific cis-regulatory elements. **b,** Binding of CAPs to liver specific gene promoters. **c,** Number of CAPs with called peaks within 2 kb of the TSSs of 57 liver specific genes with expression levels in HepG2 shown by bar color. TPM = Transcripts Per Million. **d,** Scatterplot of all genes (black points), showing log10 TPM in HepG2 (y-axis) vs number of unique CAPs with called peak +/- 2 kb of gene TSS (x-axis). Blue line indicates linear regression through black points. Red points represent 57 liver-specific genes; red line indicates linear regression through red points. **e,** same as d, but all genes expressed below 10 TPM are removed; blue line indicates linear regression through only these >10 TPM genes. **f,** Distribution of rank percentiles for expression of 57 liver-specific genes, compared to exactly matching number of CAPs (left box, n=57 genes) and to within 5% of number of TFs (center box, n=57 genes); random rank percentile for comparison is shown in right box (n=57 genes) (Mann-Whitney two-tailed p-value < 0.0001 for both exact and 5% match when tested against random). **g,** Rank percentile of expression of all genes with specific CAP's presence compared to rank percentile of equal number of random matched genes with within 5% of same number of CAPs but without specific CAP. CAPs analyzed are PAF1 (2-tailed M-W p<0.0001, n=281 genes), ATF4 (2-tailed M-W p=0.0093, n=450 genes), POLR2AphosphoS2 (2-tailed M-W p<0.0001, n=1,149 genes), and HSF1 (2-tailed M-W p=0.0002, n=400 genes). TPM = Transcripts Per Million. Boxes indicate middle quartiles, center line drawn at median, whiskers are drawn to 5-95%.

References

79 Vernimmen, D. & Bickmore, W. A. The Hierarchy of Transcriptional Activation: From Enhancer to Promoter. *Trends Genet* **31**, 696-708, doi:10.1016/j.tig.2015.10.004 (2015).

80 Lambert, S. A., Albu, M., Hughes, T. R. & Najafabadi, H. S. Motif comparison based on similarity of binding affinity profiles. *Bioinformatics* **32**, 3504-3506, doi:10.1093/bioinformatics/btw489 (2016).

81 Najafabadi, H. S., Albu, M. & Hughes, T. R. Identification of C2H2-ZF binding preferences from ChIP-seq data using RCADE. *Bioinformatics* **31**, 2879-2881, doi:10.1093/bioinformatics/btv284 (2015).

82 Bailey, T. L. *et al.* MEME SUITE: tools for motif discovery and searching. *Nucleic Acids Res* **37**, W202-208, doi:10.1093/nar/gkp335 (2009).
83 Landolin, J. M. *et al.* Sequence features that drive human promoter function and tissue specificity. *Genome Res* **20**, 890-898, doi:10.1101/gr.100370.109 (2010).
84 Whitfield, T. W. *et al.* Functional analysis of transcription factor binding sites in human promoters. *Genome Biol* **13**, R50, doi:10.1186/gb-2012-13-9-r50 (2012).
85 Hallikas, O. *et al.* Genome-wide prediction of mammalian enhancers based on analysis of transcription-factor binding affinity. *Cell* **124**, 47-59, doi:10.1016/j.cell.2005.10.042 (2006).
86 Levo, M. *et al.* Unraveling determinants of transcription factor binding outside the core binding site. *Genome Res* **25**, 1018-1029, doi:10.1101/gr.185033.114 (2015).
87 Garton, M. *et al.* A structural approach reveals how neighbouring C2H2 zinc fingers influence DNA binding specificity. *Nucleic Acids Res* **43**, 9147-9157, doi:10.1093/nar/gkv919 (2015).
88 Hauser, K. *et al.* A human transcription factor in search mode. *Nucleic Acids Res* **44**, 63-74, doi:10.1093/nar/gkv1091 (2016).
89 Slattery, M. *et al.* Cofactor binding evokes latent differences in DNA binding specificity between Hox proteins. *Cell* **147**, 1270-1282, doi:10.1016/j.cell.2011.10.053 (2011).
90 Siggers, T., Reddy, J., Barron, B. & Bulyk, M. L. Diversification of transcription factor paralogs via noncanonical modularity in C2H2 zinc finger DNA binding. *Mol Cell* **55**, 640-648, doi:10.1016/j.molcel.2014.06.019 (2014).
91 Siggers, T. & Gordan, R. Protein-DNA binding: complexities and multi-protein codes. *Nucleic Acids Res* **42**, 2099-2111, doi:10.1093/nar/gkt1112 (2014).
92 Gertz, J. *et al.* Distinct properties of cell-type-specific and shared transcription factor binding sites. *Mol Cell* **52**, 25-36, doi:10.1016/j.molcel.2013.08.037 (2013).
93 Reddy, T. E. *et al.* Genomic determination of the glucocorticoid response reveals unexpected mechanisms of gene regulation. *Genome Res* **19**, 2163-2171, doi:10.1101/gr.097022.109 (2009).
94 Chen, X., Yu, B., Carriero, N., Silva, C. & Bonneau, R. Mocap: large-scale inference of transcription factor binding sites from chromatin accessibility. *Nucleic Acids Res* **45**, 4315-4329, doi:10.1093/nar/gkx174 (2017).
95 Garber, M. *et al.* A high-throughput chromatin immunoprecipitation approach reveals principles of dynamic gene regulation in mammals. *Mol Cell* **47**, 810-822, doi:10.1016/j.molcel.2012.07.030 (2012).
96 Wang, J. *et al.* Sequence features and chromatin structure around the genomic regions bound by 119 human transcription factors. *Genome Res* **22**, 1798-1812, doi:10.1101/gr.139105.112 (2012).
97 Schmitges, F. W. *et al.* Multiparameter functional diversity of human C2H2 zinc finger proteins. *Genome Res* **26**, 1742-1752, doi:10.1101/gr.209643.116 (2016).

98 Imbeault, M., Helleboid, P. Y. & Trono, D. KRAB zinc-finger proteins contribute to the evolution of gene regulatory networks. *Nature* **543**, 550-554, doi:10.1038/nature21683 (2017).
99 Yan, J. *et al.* Transcription factor binding in human cells occurs in dense clusters formed around cohesin anchor sites. *Cell* **154**, 801-813, doi:10.1016/j.cell.2013.07.034 (2013).
100 Baresic, M., Salatino, S., Kupr, B., van Nimwegen, E. & Handschin, C. Transcriptional network analysis in muscle reveals AP-1 as a partner of PGC-1alpha in the regulation of the hypoxic gene program. *Mol Cell Biol* **34**, 2996-3012, doi:10.1128/MCB.01710-13 (2014).
101 Fernandez, P. C. *et al.* Genomic targets of the human c-Myc protein. *Genes Dev* **17**, 1115-1129, doi:10.1101/gad.1067003 (2003).
102 Baranello, L., Kouzine, F., Sanford, S. & Levens, D. ChIP bias as a function of cross-linking time. *Chromosome Res* **24**, 175-181, doi:10.1007/s10577-015-9509-1 (2016).
103 Teytelman, L. *et al.* Impact of chromatin structures on DNA processing for genomic analyses. *PLoS One* **4**, e6700, doi:10.1371/journal.pone.0006700 (2009).
104 Li, Q., Brown, J. B., Huang, H. & Bickel, P. J. Measuring reproducibility of high-throughput experiments. *The Annals of Applied Statistics* **5**, 1752-1779, doi:10.1214/11-aoas466 (2011).
105 Cirillo, L. A. *et al.* Opening of compacted chromatin by early developmental transcription factors HNF3 (FoxA) and GATA-4. *Mol Cell* **9**, 279-289 (2002).
106 Magnani, L., Eeckhoute, J. & Lupien, M. Pioneer factors: directing transcriptional regulators within the chromatin environment. *Trends Genet* **27**, 465-474, doi:10.1016/j.tig.2011.07.002 (2011).
