# Supplementary material for: Occupancy maps of 208 chromatin-associated proteins in one human cell type
Source: Nature. 2020 Jul 29;583(7818):720–8. doi: 10.1038/s41586-020-2023-4 (PMC7398277; doi:10.1038/s41586-020-2023-4)
Supplement: Supplementary file 2 — Reporting Summary [file 41586_2020_2023_MOESM2_ESM.pdf]

nature research

Corresponding author(s): Eric M. Mendenhall and Richard M. Myers

Last updated by author(s): Nov 20, 2019

Reporting Summary

Nature Research wishes to improve the reproducibility of the work that we publish. This form provides structure for consistency and transparency in reporting. For further information on Nature Research policies, see Authors & Referees and the Editorial Policy Checklist.

Statistics

For all statistical analyses, confirm that the following items are present in the figure legend, table legend, main text, or Methods section.

n/a	Confirmed	
☐	☒	The exact sample size (*n*) for each experimental group/condition, given as a discrete number and unit of measurement
☐	☒	A statement on whether measurements were taken from distinct samples or whether the same sample was measured repeatedly
☐	☒	The statistical test(s) used AND whether they are one- or two-sided<br>*Only common tests should be described solely by name; describe more complex techniques in the Methods section.*
☒	☐	A description of all covariates tested
☐	☒	A description of any assumptions or corrections, such as tests of normality and adjustment for multiple comparisons
☐	☒	A full description of the statistical parameters including central tendency (e.g. means) or other basic estimates (e.g. regression coefficient) AND variation (e.g. standard deviation) or associated estimates of uncertainty (e.g. confidence intervals)
☐	☒	For null hypothesis testing, the test statistic (e.g. *F*, *t*, *r*) with confidence intervals, effect sizes, degrees of freedom and *P* value noted<br>*Give P values as exact values whenever suitable.*
☐	☒	For Bayesian analysis, information on the choice of priors and Markov chain Monte Carlo settings
☐	☒	For hierarchical and complex designs, identification of the appropriate level for tests and full reporting of outcomes
☐	☒	Estimates of effect sizes (e.g. Cohen's *d*, Pearson's *r*), indicating how they were calculated

Our web collection on statistics for biologists contains articles on many of the points above.

Software and code

Policy information about availability of computer code

Data collection	No software was used for data collection.
Data analysis	Python v3.5<br>R v3.5<br>Shell Env (unix, bash)<br>boost C++ libraries<br>bwa v0.7.12<br>bowtie2 v2.1.0<br>spp v1.10.1<br>idr v2.0.2<br>meme v4.11.4<br>centrimo v4.11.4<br>tomtom v4.11.4<br>samtools v1.3<br>bedtools2 v2.20.0<br>fimo v4.11.4<br>phantompeakqualtools v2.0<br>picard-tools v1.88<br>trim_galore v0.3.7<br>cutadapt v1.16<br>fastqc v0.10.1<br>deeptools v3.0.0<br>kmersvm; gkmSVM v2.0<br>pybedtools v0.7.10<br>pandas v0.20.3

numpy v1.14.0
scipy v0.19.1
scikit-learn v0.19.0
ggplot2 v3.1.0; gplots v3.0.1
dplyr v0.7.5; gtools v3.0.1
ranger v0.10.1
ComplexHeatmap v1.18.0
circlize v0.4.3
igraph v1.2.1
GraphPad Prism 8 for macOS v.8.3.0
R v.3.3.2
All code available at https://github.com/chhetribsurya/PartridgeChhetri_etal

For manuscripts utilizing custom algorithms or software that are central to the research but not yet described in published literature, software must be made available to editors/reviewers. We strongly encourage code deposition in a community repository (e.g. GitHub). See the Nature Research guidelines for submitting code & software for further information.

Data

Policy information about availability of data

All manuscripts must include a data availability statement. This statement should provide the following information, where applicable:

- Accession codes, unique identifiers, or web links for publicly available datasets
- A list of figures that have associated raw data
- A description of any restrictions on data availability

All data are available at the ENCODE portal (encodeproject.org) or at Gene Expression Omnibus under accession number GSE104247.

Field-specific reporting

Please select the one below that is the best fit for your research. If you are not sure, read the appropriate sections before making your selection.

☒ Life sciences ☐ Behavioural & social sciences ☐ Ecological, evolutionary & environmental sciences

For a reference copy of the document with all sections, see nature.com/documents/nr-reporting-summary-flat.pdf

Life sciences study design

All studies must disclose on these points even when the disclosure is negative.

Sample size	No statistical methods were used to predetermine sample size.
Data exclusions	No data were excluded.
Replication	Replicate structure of ChIP/CETCh-seq experiments are described in the manuscript. Traditional antibody ChIP-seq experiments were replicated in separate grow-ups of cells, IPed separately; these are biological replicates and technical replicates for growth, crosslinking, IP, and sequencing library construction. CETCh-seq experiments were replicated at the point of nucleofection of CRISPR components, where cells are split in two equal amounts directly after nucleofection; since these represent separate pools of pre-edited cells, these are biological replicates and technical replicates for growth, crosslinking, IP, and sequencing library construction. Final data is composed of IDR-passed reproducible reads from both experimental replicates.
Randomization	The experiments were not randomized.
Blinding	The investigators were not blinded to allocation during experiments and outcome assessment.

Reporting for specific materials, systems and methods

We require information from authors about some types of materials, experimental systems and methods used in many studies. Here, indicate whether each material, system or method listed is relevant to your study. If you are not sure if a list item applies to your research, read the appropriate section before selecting a response.

Materials & experimental systems

n/a	Involved in the study
☐	☒ Antibodies
☐	☒ Eukaryotic cell lines
☒	☐ Palaeontology
☒	☐ Animals and other organisms
☒	☐ Human research participants
☒	☐ Clinical data

Methods

n/a	Involved in the study
☐	☒ ChIP-seq
☒	☐ Flow cytometry
☒	☐ MRI-based neuroimaging

Antibodies

Antibodies used

BACH1,sc-14700,E1503,Santa Cruz Biotech,5 ug per 2e7 cells;
CUX1,sc-6327,E0709,Santa Cruz Biotech,5 ug per 2e7 cells;
SIN3B,sc-13145,B2802,Santa Cruz Biotech,5 ug per 2e7 cells;
KAT2B,3378S,1,Cell Signaling,5 ug per 2e7 cells;
POLR2AphosphoS2,ab5095,GR32890-1,Abcam,5 ug per 2e7 cells;
RFX5,200-401-194,14562,Rockland,5 ug per 2e7 cells;
SMC3,ab9263,963667,Abcam,5 ug per 2e7 cells;
FOS,sc-7202,K0810,Santa Cruz Biotech,5 ug per 2e7 cells;
HCFC1,NB100-68209,A1,Novus,5 ug per 2e7 cells;
MXI1,AF4185,ZIJ0107031,RD Systems,5 ug per 2e7 cells;
TBL1XR1,ab24550,GR340121,Abcam,5 ug per 2e7 cells;
ZNF384,HPA004051,A57874,Sigma,5 ug per 2e7 cells;
ZNF143,16618-1-AP,8059,Proteintech,5 ug per 2e7 cells;
JUN,sc-1694,C2206,Santa Cruz Biotech,5 ug per 2e7 cells;
RCOR1,sc-30189,C0806,Santa Cruz Biotech,5 ug per 2e7 cells;
CHD2,ab68301,762356,Abcam,5 ug per 2e7 cells;
SUZ12,3737BF,4,Cell Signaling,5 ug per 2e7 cells;
IRF3,sc-9082,I0908,Santa Cruz Biotech,5 ug per 2e7 cells;
BHLHE40,NB100-1800,A1,Novus,5 ug per 2e7 cells;
ARID3A,NB100-279,A1,Novus,5 ug per 2e7 cells;
BRCA1,A300-000A,2,Bethyl Labs,5 ug per 2e7 cells;
NFE2L2,sc-13032,A1711,Santa Cruz Biotech,5 ug per 2e7 cells;
JUND,sc-74,unknown,Santa Cruz Biotech,5 ug per 2e7 cells;
CEBPZ,SAB2100398,QC8343,Sigma,5 ug per 2e7 cells;
TBP,ab62126,unknown,Sigma,5 ug per 2e7 cells;
POLR2A,MMS-126R,14861301,Covance,5 ug per 2e7 cells;
MAFF,M8194,125K4837,Sigma,5 ug per 2e7 cells;
HSF1,sc-9144,unknown,Santa Cruz Biotech,5 ug per 2e7 cells;
SREBF1,sc-8984,10211,Santa Cruz Biotech,5 ug per 2e7 cells;
MAFK,ab50322,904274,Abcam,5 ug per 2e7 cells;
MAZ,ab85725,GR41711-2,Abcam,5 ug per 2e7 cells;
NR3C1,sc-1002,I0310,Santa Cruz Biotech,5 ug per 2e7 cells;
ESRRA,sc-66882,A0809,Santa Cruz Biotech,5 ug per 2e7 cells;
CEBPB,sc-150,I1010,Santa Cruz Biotech,5 ug per 2e7 cells;
BMI1,unknown,unknown,unknown,unknown;
RING1,unknown,unknown,unknown,unknown;
KDM6A,unknown,unknown,unknown,unknown;
BRD4,A301-985A50,3,Bethyl Labs,5 ug per 2e7 cells;
EZH2,39875,27210001,Active Motif,5 ug per 2e7 cells;
ASH2L,A300-489A,2,Bethyl Labs,5 ug per 2e7 cells;
KDM1A,A300-215A,1,Bethyl Labs,5 ug per 2e7 cells;
NR2C2,TR4,unknown,James Engel,5 ug per 2e7 cells;
ZNF274,H00010782-A01,060729QCS1,Abnova,5 ug per 2e7 cells;
TCF7L2,2569,2,Cell Signaling,5 ug per 2e7 cells;
MYC,sc-764,H0107,Santa Cruz Biotech,5 ug per 2e7 cells;
TAF1,sc-735,K0905,Santa Cruz Biotech,5 ug per 2e7 cells;
USF1,sc-229,A2109,Santa Cruz Biotech,5 ug per 2e7 cells;
SIN3A,sc-994,F1005,Santa Cruz Biotech,5 ug per 2e7 cells;
FOSL2,sc-604,unknown,Santa Cruz Biotech,5 ug per 2e7 cells;
RXRA,sc-553 ,C1811,Santa Cruz Biotech,5 ug per 2e7 cells;
TCF12,sc-357,F2305,Santa Cruz Biotech,5 ug per 2e7 cells;
POLR2AphosphoS5,ab5408,648628,Abcam,5 ug per 2e7 cells;
HNF4A,sc-8987,G1309,Santa Cruz Biotech,5 ug per 2e7 cells;
FOXA1,sc-6553,H1209,Santa Cruz Biotech,5 ug per 2e7 cells;
YY1,sc-281,B1010,Santa Cruz Biotech,5 ug per 2e7 cells;
ATF3,sc-188,E1410,Santa Cruz Biotech,5 ug per 2e7 cells;
SRF,sc-335,F3006,Santa Cruz Biotech,5 ug per 2e7 cells;
CTCF,sc-5916,F2906,Santa Cruz Biotech,5 ug per 2e7 cells;

HDAC2,sc-6296,G0307,Santa Cruz Biotech,5 ug per 2e7 cells;
HNF4G,sc-6558,I299,Santa Cruz Biotech,5 ug per 2e7 cells;
EP300,sc-585,E2010,Santa Cruz Biotech,5 ug per 2e7 cells;
ZBTB33,sc-23871,I0308,Santa Cruz Biotech,5 ug per 2e7 cells;
CEBPD,sc-636,C1010,Santa Cruz Biotech,5 ug per 2e7 cells;
REST,Custom,20081022MA,Anderson lab,5 ug per 2e7 cells;
SP2,sc-643,K1803,Santa Cruz Biotech,5 ug per 2e7 cells;
ZBTB7A,sc-34508,H2406,Santa Cruz Biotech,5 ug per 2e7 cells;
NFIC,sc-81335,L0808,Santa Cruz Biotech,5 ug per 2e7 cells;
MYBL2,sc-724,D2109,Santa Cruz Biotech,5 ug per 2e7 cells;
MBD4,sc-271530,H1210,Santa Cruz Biotech,5 ug per 2e7 cells;
TEAD4,sc-101184,A1811,Santa Cruz Biotech,5 ug per 2e7 cells;
NR2F2,sc-271940,I1410,Santa Cruz Biotech,5 ug per 2e7 cells;
MAX,sc-197,J0809,Santa Cruz Biotech,5 ug per 2e7 cells;
ZEB1,sc-25388,D2010,Santa Cruz Biotech,5 ug per 2e7 cells;
FOXA2,AM39828,1720001,Active Motif,5 ug per 2e7 cells;
NR1H2,61178,29111001,Active Motif,5 ug per 2e7 cells;
TFAP4,WH0007023M3,07040-7A10,Sigma,5 ug per 2e7 cells;
ZMYM3,JH39.2.2F10,20130506-RAP,CDI,5 ug per 2e7 cells;
ZHX2,GTX112232,40107,GeneTex,5 ug per 2e7 cells;
ZNF189,GTX117129,40730,GeneTex,5 ug per 2e7 cells;
RUVBL1,JH39.2.1A1,20130711.YRH,CDI,5 ug per 2e7 cells;
PROX1,61092,14511001,Active Motif,5 ug per 2e7 cells;
SOX13,WH0009580M1,10061-3E8,Sigma,5 ug per 2e7 cells;
TCF7,WH0006932M1,11181-1D2,Sigma,5 ug per 2e7 cells;
ETV4,GTX114393,40184,Genetex,5 ug per 2e7 cells;
HNF1A,GTX113850,40135,Genetex,5 ug per 2e7 cells;
GATA4,39894,26310001,Active Motif,5 ug per 2e7 cells;
CBX1,39980,11213003,Active Motif,5 ug per 2e7 cells;
CREM,WH0001390M2,11056-3B5,Sigma,5 ug per 2e7 cells;
NRF1,R157.1.3H3,20140422-DNF,CDI,5 ug per 2e7 cells;
GABPA_FLAG,F1804,SLBK1346V,Sigma,5 ug per 2e7 cells;
RAD21_FLAG,F1804,SLBK1346V,Sigma,5 ug per 2e7 cells;
USF2,F1804,SLBK1346V,Sigma,5 ug per 2e7 cells;
KLF10,F1804,SLBK1346V,Sigma,5 ug per 2e7 cells;
FOXO1,F1804,SLBK1346V,Sigma,5 ug per 2e7 cells;
ATF1_FLAG,F1804,SLBK1346V,Sigma,5 ug per 2e7 cells;
CREB1_FLAG,F1804,SLBK1346V,Sigma,5 ug per 2e7 cells;
ATF4_FLAG,F1804,SLBK1346V,Sigma,5 ug per 2e7 cells;
ZNF3_FLAG,F1804,SLBK1346V,Sigma,5 ug per 2e7 cells;
HHEX_FLAG,F1804,SLBK1346V,Sigma,5 ug per 2e7 cells;
PBX2_FLAG,F1804,SLBK1346V,Sigma,5 ug per 2e7 cells;
ZNF219_FLAG,F1804,SLBK1346V,Sigma,5 ug per 2e7 cells;
MBD1_v1_FLAG,F1804,SLBK1346V,Sigma,5 ug per 2e7 cells;
MBD1_v2_FLAG,F1804,SLBK1346V,Sigma,5 ug per 2e7 cells;
DNMT3B_FLAG,F1804,SLBK1346V,Sigma,5 ug per 2e7 cells;
TCF25_FLAG,F1804,SLBK1346V,Sigma,5 ug per 2e7 cells;
SSRP1_FLAG,F1804,SLBK1346V,Sigma,5 ug per 2e7 cells;
TGIF2_FLAG,F1804,SLBK1346V,Sigma,5 ug per 2e7 cells;
HLF_FLAG,F1804,SLBK1346V,Sigma,5 ug per 2e7 cells;
HBP1_FLAG,F1804,SLBK1346V,Sigma,5 ug per 2e7 cells;
KDM3A_FLAG,F1804,SLBK1346V,Sigma,5 ug per 2e7 cells;
FOXP1_FLAG,F1804,SLBK1346V,Sigma,5 ug per 2e7 cells;
SLC30A9_FLAG,F1804,SLBK1346V,Sigma,5 ug per 2e7 cells;
ZNF644_FLAG,F1804,SLBK1346V,Sigma,5 ug per 2e7 cells;
HOMEZ_FLAG,F1804,SLBK1346V,Sigma,5 ug per 2e7 cells;
RERE_FLAG,F1804,SLBK1346V,Sigma,5 ug per 2e7 cells;
SAP130_FLAG,F1804,SLBK1346V,Sigma,5 ug per 2e7 cells;
KLF11_FLAG,F1804,SLBK1346V,Sigma,5 ug per 2e7 cells;
KMT2B_FLAG,F1804,SLBK1346V,Sigma,5 ug per 2e7 cells;
NR2F6_FLAG,F1804,SLBK1346V,Sigma,5 ug per 2e7 cells;
ARID4B_FLAG,F1804,SLBK1346V,Sigma,5 ug per 2e7 cells;
GATAD1_FLAG,F1804,SLBK1346V,Sigma,5 ug per 2e7 cells;
ZNF792_FLAG,F1804,SLBK1346V,Sigma,5 ug per 2e7 cells;
ZNF652_FLAG,F1804,SLBK1346V,Sigma,5 ug per 2e7 cells;
GATAD2A_FLAG,F1804,SLBK1346V,Sigma,5 ug per 2e7 cells;
NCoA2_FLAG,F1804,SLBK1346V,Sigma,5 ug per 2e7 cells;
TEAD1_FLAG,F1804,SLBK1346V,Sigma,5 ug per 2e7 cells;
NFYC_FLAG,F1804,SLBK1346V,Sigma,5 ug per 2e7 cells;
CEBPG_FLAG,F1804,SLBK1346V,Sigma,5 ug per 2e7 cells;
KLF9_FLAG,F1804,SLBK1346V,Sigma,5 ug per 2e7 cells;
DRAP1_FLAG,F1804,SLBK1346V,Sigma,5 ug per 2e7 cells;
MLX_FLAG,F1804,SLBK1346V,Sigma,5 ug per 2e7 cells;
ZNF511_FLAG,F1804,SLBK1346V,Sigma,5 ug per 2e7 cells;
MIXL1_FLAG,F1804,SLBK1346V,Sigma,5 ug per 2e7 cells;

ZSCAN9_FLAG,F1804,SLBK1346V,Sigma,5 ug per 2e7 cells;
NR2F1_FLAG,F1804,SLBK1346V,Sigma,5 ug per 2e7 cells;
TFE3_FLAG,F1804,SLBK1346V,Sigma,5 ug per 2e7 cells;
KAT8_FLAG,F1804,SLBK1346V,Sigma,5 ug per 2e7 cells;
RXRB_FLAG,F1804,SLBK1346V,Sigma,5 ug per 2e7 cells;
SOX5_FLAG,F1804,SLBK1346V,Sigma,5 ug per 2e7 cells;
KLF16_FLAG,F1804,SLBK1346V,Sigma,5 ug per 2e7 cells;
KLF6_v2_FLAG,F1804,SLBK1346V,Sigma,5 ug per 2e7 cells;
THAP11_FLAG,F1804,SLBK1346V,Sigma,5 ug per 2e7 cells;
FOXA3_FLAG,F1804,SLBK1346V,Sigma,5 ug per 2e7 cells;
ELF3_FLAG,F1804,SLBK1346V,Sigma,5 ug per 2e7 cells;
ZBTB26_FLAG,F1804,SLBK1346V,Sigma,5 ug per 2e7 cells;
TEAD3_FLAG,F1804,SLBK1346V,Sigma,5 ug per 2e7 cells;
GABPB1_v2_FLAG,F1804,SLBK1346V,Sigma,5 ug per 2e7 cells;
ERF_FLAG,F1804,SLBK1346V,Sigma,5 ug per 2e7 cells;
KAT7_FLAG,F1804,SLBK1346V,Sigma,5 ug per 2e7 cells;
MXD3_v1_FLAG,F1804,SLBK1346V,Sigma,5 ug per 2e7 cells;
ZNF580_FLAG,F1804,SLBK1346V,Sigma,5 ug per 2e7 cells;
CIZ1_FLAG,F1804,SLBK1346V,Sigma,5 ug per 2e7 cells;
MIER3_FLAG,F1804,SLBK1346V,Sigma,5 ug per 2e7 cells;
HMGXB4_FLAG,F1804,SLBK1346V,Sigma,5 ug per 2e7 cells;
ZGPAT_FLAG,F1804,SLBK1346V,Sigma,5 ug per 2e7 cells;
RARA_FLAG,F1804,SLBK1346V,Sigma,5 ug per 2e7 cells;
ARID5B_FLAG,F1804,SLBK1346V,Sigma,5 ug per 2e7 cells;
MXD4_FLAG,F1804,SLBK1346V,Sigma,5 ug per 2e7 cells;
CEBPA_FLAG,F1804,SLBK1346V,Sigma,5 ug per 2e7 cells;
ZFP1_v1_FLAG,F1804,SLBK1346V,Sigma,5 ug per 2e7 cells;
NFIL3_FLAG,F1804,SLBK1346V,Sigma,5 ug per 2e7 cells;
SP5_FLAG,F1804,SLBK1346V,Sigma,5 ug per 2e7 cells;
TFDP1_FLAG,F1804,SLBK1346V,Sigma,5 ug per 2e7 cells;
RFXANK_FLAG,F1804,SLBK1346V,Sigma,5 ug per 2e7 cells;
DMAP1_FLAG,F1804,SLBK1346V,Sigma,5 ug per 2e7 cells;
THRB_FLAG,F1804,SLBK1346V,Sigma,5 ug per 2e7 cells;
PPARG_v1_FLAG,F1804,SLBK1346V,Sigma,5 ug per 2e7 cells;
HMG20B_v2_FLAG,F1804,SLBK1346V,Sigma,5 ug per 2e7 cells;
PAF1_v1_FLAG,F1804,SLBK1346V,Sigma,5 ug per 2e7 cells;
MIER2_FLAG,F1804,SLBK1346V,Sigma,5 ug per 2e7 cells;
NFIA_v1_FLAG,F1804,SLBK1346V,Sigma,5 ug per 2e7 cells;
RCOR2_FLAG,F1804,SLBK1346V,Sigma,5 ug per 2e7 cells;
GMEB2_FLAG,F1804,SLBK1346V,Sigma,5 ug per 2e7 cells;
ZKSCAN8_FLAG,F1804,SLBK1346V,Sigma,5 ug per 2e7 cells;
HMG20A_FLAG,F1804,SLBK1346V,Sigma,5 ug per 2e7 cells;
ZNF48_FLAG,F1804,SLBK1346V,Sigma,5 ug per 2e7 cells;
UBP1_FLAG,F1804,SLBK1346V,Sigma,5 ug per 2e7 cells;
MTA1_FLAG,F1804,SLBK1346V,Sigma,5 ug per 2e7 cells;
ZFP64_FLAG,F1804,SLBK1346V,Sigma,5 ug per 2e7 cells;
FOXK1_FLAG,F1804,SLBK1346V,Sigma,5 ug per 2e7 cells;
RFX3_iso1_FLAG,F1804,SLBK1346V,Sigma,5 ug per 2e7 cells;
ZNF7_iso2_FLAG,F1804,SLBK1346V,Sigma,5 ug per 2e7 cells;
SOX13_iso1_FLAG,F1804,SLBK1346V,Sigma,5 ug per 2e7 cells;
SMAD4_iso1_FLAG,F1804,SLBK1346V,Sigma,5 ug per 2e7 cells;
BCL6_iso1_FLAG,F1804,SLBK1346V,Sigma,5 ug per 2e7 cells;
ZNF331_FLAG,F1804,SLBK1346V,Sigma,5 ug per 2e7 cells;
THRA_iso1_FLAG,F1804,SLBK1346V,Sigma,5 ug per 2e7 cells;
SIX4_iso1_FLAG,F1804,SLBK1346V,Sigma,5 ug per 2e7 cells;
ZHX3_iso1_FLAG,F1804,SLBK1346V,Sigma,5 ug per 2e7 cells;
ZNF544_iso1_FLAG,F1804,SLBK1346V,Sigma,5 ug per 2e7 cells;
ZNF334_iso1_FLAG,F1804,SLBK1346V,Sigma,5 ug per 2e7 cells;
ZNF281_FLAG,F1804,SLBK1346V,Sigma,5 ug per 2e7 cells;
NPAS2_iso2_FLAG,F1804,SLBK1346V,Sigma,5 ug per 2e7 cells;
ZSCAN29_iso1_FLAG,F1804,SLBN5629V,Sigma,5 ug per 2e7 cells;
E2F7_iso1_FLAG,F1804,SLBN5629V,Sigma,5 ug per 2e7 cells;
PRDM10_FLAG,F1804,SLBN5629V,Sigma,5 ug per 2e7 cells;
KDM2A_FLAG,F1804,SLBN5629V,Sigma,5 ug per 2e7 cells;
PAXIP1_iso1_FLAG,F1804,SLBN5629V,Sigma,5 ug per 2e7 cells;
JARID2_iso1_FLAG,F1804,SLBN5629V,Sigma,5 ug per 2e7 cells;
RREB1_iso2_FLAG,F1804,SLBN5629V,Sigma,5 ug per 2e7 cells;
ZBTB21_FLAG,F1804,SLBN5629V,Sigma,5 ug per 2e7 cells;
ZC3H4_FLAG,F1804,SLBN5629V,Sigma,5 ug per 2e7 cells;
SP1_FLAG,F1804,SLBN5629V,Sigma,5 ug per 2e7 cells;
CBX5_FLAG,F1804,SLBN5629V,Sigma,5 ug per 2e7 cells;
ZNF12_FLAG,F1804,SLBN5629V,Sigma,5 ug per 2e7 cells;
ZNF335_FLAG,F1804,SLBN5629V,Sigma,5 ug per 2e7 cells;
HMGXB3_FLAG,F1804,SLBN5629V,Sigma,5 ug per 2e7 cells;

ZCCHC11_FLAG,F1804,SLBN5629V,Sigma,5 ug per 2e7 cells;

Validation

Primary characterization by Western blot or Immunoprecipitation/blot
For every TF ChIP-seq antibody, ENCODE data producers first perform an immunoblot characterization. This can be either a standard Western blot, or an immunoprecipitation followed by a Western blot ("IP Western"). If the blot results do not meet the parameters and thresholds given below, then Primary Characterization Method 2 (IP mass spec) is performed. In the latter case, the failed (or partially failed) immunoblot that preceded IP/mass spec is included in the report so that researchers and data users can independently evaluate the data for each antibody.
Immunoblot parameters:
a. ENCODE developed a set of working parameters and thresholds to identify antibodies with a high likelihood of being specific for the target factor. The parameters allow for modest variation in gel migration characteristics and in band number to accommodate known behaviors of typical nuclear factors. Current acceptable parameters are that the major band is within 20% of the size predicted by the size of the coding region and corresponds to >50% of all bands on the gel (excluding the antibody bands in the case of an immunoprecipitation). If the western or IP-western results meet these criteria, we consider the antibody to meet expectations for the primary characterization. The immunoblot results (which must include appropriate size markers) are submitted as evidence for each cell type or tissue tested. For IP-westerns, a control IgG precipitation is also performed and analyzed on the same gel.
b. If the antibody fails to pass the immunoblot tests because the bands observed are too numerous, or too far from the predicted migration behavior, it can be “rescued” by a secondary characterization that supports the conclusion that the band(s) detected correspond to the correct protein (e.g. all bands are reduced by treatment with a specific siRNA to that protein; see secondary characterizations).
c. If the antibody passes the immunoblot tests, a further characterization is required to support the successful immunoblot. This can be Primary Characterization Method 2 (IP mass spec) or any one of the Secondary Characterization methods in IB.

Primary Characterization Method 2: Immunoprecipitation followed by mass spectrometry ("IP mass spec")
If the immunoblot characterization data was not successful (ranging from no bands to patterns that do not meet the thresholds given above), then Mass spec of an immunoprecipitation can be performed. The failed or ambiguous immunoblot is, however, shown as part of the antibody characterization dataset. Because the IP/mass spec assay provides explicit evidence about the identity of the TF detected, it can also be used in lieu of Secondary methods after a successful Immunoblot (see flowchart above).
For TF mass spec, a cell or nuclear extract is immunoprecipitated with the same antibody used to perform ChIP-seq. That IP is then fractionated on a denaturing polyacrylamide gel, and the fractions are prepared and analyzed by mass spec as described below.
What is reported for IP mass spec:
• IP-western blot of gel image with outline of gel slices submitted for mass spec.
• All peptides (with peptide counts) from all immunoreactive bands.
• Fold enrichment of all peptides in the immunoreactive bands vs either mock IP or a set of proteins that have been immunoprecipitated from the same cell type using a collection of other antibodies from the same host species (the list of proteins used as the set of IP contaminants list must be provided).
• Indication as to which proteins above the target protein on the ranked list (ranked by fold enrichment) are TFs and which TFs are members of the same TF family as the target protein.
IP mass spec requirements to be considered fully validated for ENCODE data:
• The target protein should be enriched in the IP when compared to a mock IP or to a set of proteins that have been immunoprecipitated from the same cell type using a collection of other antibodies from the same host species.
• The target should be in the top 25 ranked proteins and the top most-enriched TF (by fold enrichment) in the immunoreactive band, unless the higher ranked TFs are known interacting partners of the target TF and/or a known interacting partner of one of the other higher ranked TFs that is a known partner of the target TF. Evidence for interaction can come from publications or refer to records in interaction databases such as BioGRID, or other sources..
• The target should be the top ranked member of that family of TFs (exceptions will be allowed if a publication is provided that demonstrates that a higher ranked family member is known to dimerize with the target protein).
• In situations for which the target protein has 0 peptides in the mock IP, a ranking by enrichment can not be performed. In that case, the following criteria are used:
• If the target TF is the top TF as ranked by number of detected peptides, then the antibody passes this characterization method.
• If the target TF is not the top TF but the TFs having more counts have previously been documented to be in the same complex and/or interact directly with the target TF, then the antibody passes this characterization method.
• If the target TF is not the top TF but the non-target TFs (having a greater number of detected peptides) were detected using mass spec analysis of two different 4 antibodies to the target TF, then the antibody passes this characterization method (with the assumption that the other TFs are bona fide interacting TFs that have not yet been documented in the literature).
• If the target TF is not the top TF and the TFs having more counts have never been linked to the target TF then this antibody is flagged, with the explanation that enrichment could not be determined due to the lack of detected peptides in the IgG and that

no published data exists linking the target to the non-target TFs.

• If an antibody doesn't meet these characteristics, the antibody characterization document can be submitted for consideration as a special request (see Note 3).

Additional situations for Primary Characterization
a. Guidelines for using the same lot number of a previously characterized antibody in a new cell type
If a specific lot number for an antibody has previously passed characterization in another cell type, and if the banding pattern on the immunoblot or immunoprecipitation is the same in the new cell type as in the characterized cell type, then no further characterization is needed for the antibody in that new cell type. If the banding pattern is different in the new cell type, a secondary characterization is performed in the new cell type. Exceptions to this guideline will be considered for studies of human tissues (due to the fact that it is often hard to obtain sufficient tissue for the antibody characterization and a ChIP-seq experiment). If an antibody has passed characterization criteria in 2 different human cell lines and/or tissues, it does not have to be characterized in each tissue type.
b. Guidelines for using a different lot number of a previously characterized antibody
For the first time that a new lot number is used for a previously-characterized antibody, a Primary Characterization method (immunoblot or IP mass spec) is performed with one of the same cell types used to characterize the previous lot number plus the cell type for which ChIPseq data will be deposited for the new lot number; the ENCODE antibody accession number of the specific previously characterized lot that should be used for comparison is indicated. If the patterns for the new lot number are the same in the previously characterized cell type and in the cell type for which ChIP-seq data will be deposited as shown in the characterization of the original lot number of that antibody, then no further characterization is required. If the banding patterns are different, a secondary characterization is performed. Exceptions to this guideline will be considered for analysis of tissues with antibodies that have been well-characterized and used extensively by the field (e.g a monoclonal antibody to RNAPII). In this case, if a previous lot number of an antibody has passed characterization criteria in 2 different human cell lines and/or tissues, the new lot number does not have to be characterized in each tissue type.
Other primary characterization methods. If other methods not specified above are used for primary characterization of an antibody, the antibody characterization document is submitted as a special request and is so annotated and flagged.

IB. Secondary Characterization Methods. These methods are used to support and clarify the Immunoblot data. In particular, they aim to verify that a band or bands observed on the prior blot correspond to the intended TF. At least one successful Secondary Characterization (or alternatively IP/Mass spec as shown in figure 1 above) is required to support a successful Western or IP/Western.
Secondary Characterization Method 1: siRNA or shRNA against the mRNA of the target protein
For siRNA or shRNA knockdown characterization, the band(s) detected by the antibody on a western blot should be reduced by at least 50% of the control signal. These methods are especially intended to address instances where the Western or IP Western data give multiple bands and unpredicted migration patterns. The sequence or vendor and catalog number of the oligonucleotide(s) reagent should be provided. A control knockdown should also be performed. Cell types will be labeled and size markers should be included on the immunoblot. A brief description of the transfection protocol will also be provided.
Secondary Characterization Method 2: ChIP-seq data from a previously characterized antibody
If ChIP-seq data for a different lot number of a previously characterized antibody or a previously characterized, but different, antibody for a given transcriptional regulator is available, this ChIPseq data can be used to evaluate a new antibody or new lot number. The ChIP-seq data from the new antibody or new lot number are compared to the previous ChIP-seq using IDR. If the two datasets pass the ENCODE IDR cuts-offs for narrow peak ChIP-seq reproducibility (see below for current IDR standards), then the secondary characterization of the new antibody/lot number is scored as successful. For data submission, the specific antibody lot and ChIP-seq data used for the comparison are identified by their ENCODE antibody and experiment accessions, respectively. In a similar way, ChIP-seq data obtained using an endogenous epitope-tagged version of the target protein can be used for comparison.
Secondary Characterization Method 3: Expression patterns of an epitope-tagged transcription factor
Especially useful for TFs that are resistant to knockdown using shRNA or siRNAs (e.g. very stable proteins) is a secondary characterization method that involves comparison to overexpressed or endogenously epitope-tagged TF proteins. In this case, the primary characterization of the TF antibody must first show the appropriate specificity on the western or IP-western. Then, two side-by-side immunoblots can be performed using control cells and cells expressing the tagged-factor. The first immunoblot employs the antibody to the tag to show the position of the exogenous factor band(s) and the second immunoblot employs the antibody to the endogenous factor to show that the band(s) in the control and ectopically expressing cases correspond.

Secondary Characterization Method 4: Motif analysis
Motif enrichment for antibody characterization requires pre-existing information about the DNA

sequence to which the factor binds. Enrichment of a known motif for a target TF in a ChIP experiment is evidence that the antibody does in fact recognize the target TF.
Motif enrichment can be used as a validation method for antibodies that meet the following criteria:
i. The antibody under consideration binds a sequence-specific transcription factor
ii. The DNA motif sequence bound by the transcription factor has been previously wellcharacterized by either in vitro or in vivo experiments
iii. The antibody is raised to a unique region of the transcription factor (in relation to other TFs in the same family)
Motif analysis can be performed using high-quality peaks (0.01 IDR cut off) from the ChIP experiment. Proper use of motif enrichment analysis for antibody validation should include metrics indicative of:
i. Global Enrichment z-score: Enrichment of the motif sequence in the ChIP peak over shuffled randomized motifs of the same sequence composition
ii. Positional Bias z-score: A measure of the distance of the motif to the peak center
iii. Peak Rank Bias z-score: A measure of the distribution of the motif in peaks ranked by ChIP intensity
The mean of these three z-scores is used in computing the final enrichment rank among 282 motif groups, as well as the “accept probability”. The “accept probability” is a combined metric that measures confidence in the antibody under investigation being of high quality for ChIP experiments. An accept probability greater that 0.6 is the current criteria for accepting an antibody as passing secondary characterization by motif enrichment (see note 4)..
The Characterization report where Motif enrichment is used for antibody secondary validations includes:
i. The ENCODE DCC file identifiers for the peaks files (.bed files) used in the analysis
ii. A brief description of the analysis method and a reference to the standards documents
iii. The accept probability score from the motif analysis pipeline
iv. The identified motif (PWM) and its enrichment rank
v. The positional bias score as well as the peak rank score
Motif analysis cannot be used when:
i. The transcription factor does not bind in a sequence-specific manner
ii. There is no information for the DNA motif bound by the TF
iii. When it has been shown that the TF bind to DNA indirectly by interacting with other proteins that directly bind DNA

Because transcription factors are recruited by multiple mechanisms, failure of a data set to meet the motif enrichment criteria does not indicate poor antibody quality or poor data quality. Such antibodies can be validated using other Secondary Characterization methods.
Additional notes on methods for antibody characterization:
1. These methods refer to characterization of antibodies that recognize endogenously expressed proteins. The requirements for characterization of antibodies that recognize epitope-tagged proteins are described elsewhere.
2. Current IDR standards for a narrow-peak ChIP-seq dataset are: Rescue Ratio RR_new = | Np U Nt | / | Np ^ Nt | Self consistency ratio SR_new = | N1 U N2 | / | N1 ^ N2 | where ^ = intersection (common) of 2 peak sets U = union (merge) of 2 peaks sets
If (R_new > 2) AND (SR_new > 2) then the replicates are proclaimed to have low reproducibility (failed) and flagged with -1 quality score If (RR_new > 2) OR (SR_new > 2) but not both, then the replicates are proclaimed to have moderate reproducibility (passed) and flagged with a 0 quality score If (RR_new <= 2) AND (SR_new <= 2) then the replicates are proclaimed to have high reproducibility (passed) and flagged with +1 quality score.
3. Scientists within and outside ENCODE have learned over time that some antibodies that perform well in ChIP assays nevertheless fail to pass the conventional tests that comprise primary and secondary analyses. Therefore, exceptions to the basic characterization can be considered for such cases. The antibody characterization review committee together with the ENCODE Production PIs, will consider these on a case by case basis. Datasets using such reagents, referred to as “exempt” antibodies, will be flagged in the ENCODE data.

Guidelines for ENCODE Epitope-tagged transcription factor ChIP-seq
ENCODE uses a variety of methods to characterize tagged TFs in ChIP-seq experiments, and these methods are categorized as being either genomic characterizations (to ensure the correct locus of interest was tagged properly), or immunological characterizations (to ensure the antibody recognizes the epitope-tagged protein) Typically, one form of the experiments listed under part A (Genomic DNA Characterization) and one form of the experiments listed under part B (Immunocharacterization) is used for a given TF.

A. Genomic DNA characterization (A-1 or A-2 should be performed)
The experimental design relies on correct integration of the epitope tag sequence into genomic DNA of the recipient cell line. One of the following genomic characterizations is performed:
A-1. PCR analysis
PCR is used to verify the presence of the intended integrated sequence at the intended site of integration. PCR primers are designed such that the amplification product is generated only if the epitope tag is integrated correctly in the genomic DNA. In this design, one primer is selected to anneal outside the region used for the homology-directed repair (the mechanism used for integration), and one primer is located inside the tag sequence.
A-2. DNA sequencing of integrated tag segment

Genomic DNA is used to show epitope-tag integration at the designed target site. Sanger or next-generation DNA sequencing of genomic DNA showing correct integration of the tag sequence is performed for this determination.

What is reported for Genomic Characterizations:
A gel image of the PCR reaction products with a DNA sizing ladder. A negative control sample (amplification from wild-type DNA) should be included if available. The expected size should be indicated, along with the PCR primer sequences and thermocycling conditions used to generate the products. For sequencing data, an electropherogram (Sanger sequence trace) or genome browser screenshot with an indication of the integration region within the wild-type genomic DNA.
Genomic Characterization requirements to be considered fully validated for ENCODE data:
Ideally for PCR and sequencing data, results from both replicates should be represented. If, however, only one replicate is present or passes genomic validation, then a passing grade can be assigned if both replicates passed IDR from ChIP-seq.

B. Immunocharacterization (B-1 or B2 should be performed)
The epitope-tagged ChIP-seq experiment relies on a well-characterized antibody raised against the epitope tag. Immunological characterization of the antibody in each parental target cell population or type, prior to introduction of the tag, is performed. This characterization is used to detect any significant off-target ChIP signals due to cross-reactivity of the antibody with proteins other than the designed tagged protein. Epitope-tagged cell immunocharacterization is done by performing one of the methods below (B-1 or B-2).
B-1. Immunoblot (Western blot) or Immunoprecipitation blot (IP-Western blot)
It is preferred that the antibody used for the blots is the same one as used in the ChIP-seq experiment. However, it is recognized that antibodies differ in their ability to detect denatured and native proteins. Therefore, if necessary, another antibody raised against the epitope tag can be used for the Western blot. A band (or bands) corresponding to predicted migration for the epitope tagged protein (or multiple forms, if they are predicted) should be visible when comparing the epitope-tagged cell line versus the “wild-type” cell line. The background control for immunocharacterization is the “wild type” cell line without a tag integration event. This control experiment is performed at least once for each parental cell line that is used.
What is reported for Immunoblot or Immunoprecipitation blot (IP-Western blot):
An image of the blot/gel showing affinity of the antibody for the epitope-tagged protein from either cell lysates (immunoblot) or immunoprecipitated proteins from cell lysates (immunoprecipitation blot). A protein sizing ladder should be included as well as a description of the blotting method and conditions for immunostaining. For immunoprecipitation blots, the antibodies used for both immunoprecipitation and visualization should be indicated. The expected size of the tagged target protein should be indicated as well as other bands that might correspond to either lower size degradation products or putative post-translational modifications.
Immunoblot or Immunoprecipitation blot requirements to be considered fully validated for ENCODE data:
The protein band of interest must be within 20% of the size predicted by the coding region. If the Western blot or IP-Western blot result meets this criteria, we consider the engineered cell line to meet expectations. If however, protein sizes do not match expected sizes which include the tag, then Western blots with native antibodies from commercial vendors can be used for compliance if the sizes are equivalent. Protein modifications and degradation products are known to complicate the sizing and intensity of bands, therefore, all instances must be thoroughly explained in the corresponding captions so that users of the data are made aware.
B-2. Immunoprecipitation followed by mass spectrometry
A cell or nuclear extract from cells expressing the tagged protein is immunoprecipitated with the same antibody used to perform ChIP-seq. These characterizations should be performed using the same lot number of antibody as used in the reported ChIP-seq experiments. The IP product is then fractionated on a denaturing polyacrylamide gel, and the fractions are prepared and analyzed by mass spec as described below.
What is reported for IP mass spec:
An IP-Western blot gel image with an outline of gel slices that were submitted for mass spec should be reported. If, however, the entire IP was used for the mass spec analysis, a Western blot or IP-Western blot image is not required. A list of all peptides (with peptide counts) from all immunoreactive bands should be presented in tabular format. Fold enrichment of all the peptides in the immunoreactive bands vs either mock IP or a set of proteins that have been immunoprecipitated from the same cell type using a collection of other antibodies from the same host species (the list of proteins used as the set of IP contaminants list must be provided) should also be determined.
IP mass spec requirements to be considered fully validated for ENCODE data:
The target protein should be enriched within the top 20 ranked proteins in the IP when compared to a mock IP or to a set of proteins that have been immunoprecipitated from the same cell type using a collection of other antibodies from the same host species. Ideally, the target TF would represent the highest ranking TF within this enrichment. If it is not however, then the production lab should indicate potential complexes or interacting partners (if known) that have co-immunoprecipitated with their target TF or provide an appropriate audit if the ChIP-seq data is deemed of high quality. In situations involving mock IPs for which the target protein has 0 peptides in the mock IP, a ranking by enrichment cannot be performed. In this case, the following criteria are considered for validation: the target TF is the top TF present as ranked by

the number of detected peptides or, the target TF is not the top TF ranked by peptide counts but is documented to be in a complex or have interactions with the other TFs having more counts. For situations where the target TF is not the top TF and there are no documented instances of interactions with other TFs having more counts, then an audit is assigned with the explanation that the enrichment could not be determined due to the lack of detected peptides in the IgG control and that no published data exists linking the target to the non-target TFs.

EXCEPTIONS
We realize that, in some cases, situations may arise in which antibodies or tagged factor lines do not pass the above standards, but the data producers feel that the datasets should be made available to users. Often there is data from other sources that support a ChIP-seq dataset that has not passed both A and B standards. Examples include the same epitope tagging reagents having passed in another cell type, or a high overlap of peaks to an antibody based dataset in the same cell type, or a highly similar motif found to one previously published for that factor. Therefore, exceptions to these characterization standards are considered for special cases. The antibody characterization review committee of the ENCODE Consortium will consider each special request. If an exception is granted, the datasets using these "exempt" antibodies will be flagged in the ENCODE datasets.

Eukaryotic cell lines

Policy information about cell lines

Cell line source(s)	HepG2 - ATCC - HB-8065
Authentication	Phenotypic characterization.
Mycoplasma contamination	Cells are routinely tested for Mycoplasma contamination. All tests were negative.
Commonly misidentified lines (See ICLAC register)	HepG2 is not listed as being commonly misidentified.

ChIP-seq

Data deposition

☒ Confirm that both raw and final processed data have been deposited in a public database such as GEO.

☒ Confirm that you have deposited or provided access to graph files (e.g. BED files) for the called peaks.

Data access links
May remain private before publication.

encodeproject.org
GEO: GSE104247

Files in database submission

GSM2797484 ARID3A
GSM2797485 ARID4B
GSM2797486 ARID5B
GSM2797487 ASH2L
GSM2797488 ATF1
GSM2797489 ATF3
GSM2797490 ATF4
GSM2797491 BACH1
GSM2797492 BCL6_iso1
GSM2797493 BHLHE40
GSM2797494 BMI1
GSM2797495 BRCA1
GSM2797496 BRD4
GSM2797497 CBX1
GSM2797498 CBX5
GSM2797499 CEBPA
GSM2797500 CEBPB
GSM2797501 CEBPD
GSM2797502 CEBPG
GSM2797503 CEBPZ
GSM2797504 CHD2
GSM2797505 CIZ1
GSM2797506 CREB1
GSM2797507 CREM
GSM2797508 CTCF
GSM2797509 CUX1
GSM2797510 DMAP1
GSM2797511 DNMT3B
GSM2797512 DRAP1
GSM2797513 E2F7_iso1

GSM2797514	ELF3
GSM2797515	EP300
GSM2797516	ERF
GSM2797517	ESRRA
GSM2797518	ETV4
GSM2797519	EZH2
GSM2797520	FOS
GSM2797521	FOSL2
GSM2797522	FOXA1
GSM2797523	FOXA2
GSM2797524	FOXA3
GSM2797525	FOXK1
GSM2797526	FOXO1
GSM2797527	FOXP1
GSM2797528	GABPA
GSM2797529	GABPB1_v2
GSM2797530	GATA4
GSM2797531	GATAD1
GSM2797532	GATAD2A
GSM2797533	GMEB2
GSM2797534	HBP1
GSM2797535	HCFC1
GSM2797536	HDAC2
GSM2797537	HHEX
GSM2797538	HLF
GSM2797539	HMG20A
GSM2797540	HMG20B_v2
GSM2797541	HMGXB3
GSM2797542	HMGXB4
GSM2797543	HNF1A
GSM2797544	HNF4A
GSM2797545	HNF4G
GSM2797546	HOMEZ
GSM2797547	HSF1
GSM2797548	IRF3
GSM2797549	JARID2_iso1
GSM2797550	JUND
GSM2797551	JUN
GSM2797552	KAT2B
GSM2797553	KAT7
GSM2797554	KAT8
GSM2797555	KDM1A
GSM2797556	KDM2A
GSM2797557	KDM3A
GSM2797558	KDM6A
GSM2797559	KLF10
GSM2797560	KLF11
GSM2797561	KLF16
GSM2797562	KLF6_v2
GSM2797563	KLF9
GSM2797564	KMT2B
GSM2797565	MAFF
GSM2797566	MAFK
GSM2797567	MAX
GSM2797568	MAZ
GSM2797569	MBD1_v1
GSM2797570	MBD1_v2
GSM2797571	MBD4
GSM2797572	MIER2
GSM2797573	MIER3
GSM2797574	MIXL1
GSM2797575	MLX
GSM2797576	MTA1
GSM2797577	MXD3_v1
GSM2797578	MXD4
GSM2797579	MXI1
GSM2797580	MYBL2
GSM2797581	MYC
GSM2797582	NCoA2
GSM2797583	NFE2L2
GSM2797584	NFIA_v1
GSM2797585	NFIC
GSM2797586	NFIL3
GSM2797587	NFYC
GSM2797588	NPAS2_iso2

GSM2797589	NR1H2
GSM2797590	NR2C2
GSM2797591	NR2F1
GSM2797592	NR2F2
GSM2797593	NR2F6
GSM2797594	NR3C1
GSM2797595	NRF1
GSM2797596	PAF1_v1
GSM2797597	PAXIP1_iso1
GSM2797598	PBX2
GSM2797599	POLR2A
GSM2797600	POLR2AphosphoS2
GSM2797601	POLR2AphosphoS5
GSM2797602	PPARG_v1
GSM2797603	PRDM10
GSM2797604	PROX1
GSM2797605	RAD21
GSM2797606	RARA
GSM2797607	RCOR1
GSM2797608	RCOR2
GSM2797609	RERE
GSM2797610	REST
GSM2797611	RFX3_iso1
GSM2797612	RFX5
GSM2797613	RFXANK
GSM2797614	RING1
GSM2797615	RREB1_iso2
GSM2797616	RUVBL1
GSM2797617	RXRA
GSM2797618	RXRB
GSM2797619	SAP130
GSM2797620	SIN3A
GSM2797621	SIN3B
GSM2797622	SIX4_iso1
GSM2797623	SLC30A9
GSM2797624	SMAD4_iso1
GSM2797625	SMC3
GSM2797626	SOX13
GSM2797627	SOX13_iso1
GSM2797628	SOX5
GSM2797629	SP1
GSM2797630	SP2
GSM2797631	SP5
GSM2797632	SREBF1
GSM2797633	SRF
GSM2797634	SSRP1
GSM2797635	SUZ12
GSM2797636	TAF1
GSM2797637	TBL1XR1
GSM2797638	TBP
GSM2797639	TCF12
GSM2797640	TCF25
GSM2797641	TCF7
GSM2797642	TCF7L2
GSM2797643	TEAD1
GSM2797644	TEAD3
GSM2797645	TEAD4
GSM2797646	TFAP4
GSM2797647	TFDP1
GSM2797648	TFE3
GSM2797649	TGIF2
GSM2797650	THAP11
GSM2797651	THRA_iso1
GSM2797652	THRB
GSM2797653	UBP1
GSM2797654	USF1
GSM2797655	USF2
GSM2797656	YY1
GSM2797657	ZBTB21
GSM2797658	ZBTB26
GSM2797659	ZBTB33
GSM2797660	ZBTB7A
GSM2797661	ZC3H4
GSM2797662	ZCCHC11
GSM2797663	ZEB1

GSM2797664	ZFP1_v1
GSM2797665	ZFP64
GSM2797666	ZGPAT
GSM2797667	ZHX2
GSM2797668	ZHX3_iso1
GSM2797669	ZKSCAN8
GSM2797670	ZMYM3
GSM2797671	ZNF12
GSM2797672	ZNF143
GSM2797673	ZNF189
GSM2797674	ZNF219
GSM2797675	ZNF274
GSM2797676	ZNF281
GSM2797677	ZNF331
GSM2797678	ZNF334_iso1
GSM2797679	ZNF335
GSM2797680	ZNF384
GSM2797681	ZNF3
GSM2797682	ZNF48
GSM2797683	ZNF511
GSM2797684	ZNF544_iso1
GSM2797685	ZNF580
GSM2797686	ZNF644
GSM2797687	ZNF652
GSM2797688	ZNF792
GSM2797689	ZNF7_iso2
GSM2797690	ZSCAN29_iso1
GSM2797691	ZSCAN9
GSM2797692	Input 1
GSM2797693	Input 2
GSM2797694	Input 3
GSM2797695	Input 4
GSM2797696	Input 5
GSM2797697	Input 6
GSM2797698	Input 7
GSM2797699	Input 8
GSM2797700	Input 9
GSM2797701	Input 10
GSM2797702	Input 11
GSM2797703	Input 12
GSM2797704	Input 13
GSM2797705	Input 14
GSM2797706	Input 15
GSM2797707	Input 16
GSM2797708	Input 17
GSM2797709	Input 18
GSM2797710	Input 19
GSM2797711	Input 20
GSM2797712	Input 21
GSM2797713	Input 22
GSM2797714	Input 23
GSM2797715	Input 24
GSM2797716	Input 25
GSM2797717	Input 26
GSM2797718	Input 27
GSM2797719	Input 28
GSM2797720	Input 29
GSM2797721	Input 30
GSM2797722	Input 31
GSM2797723	Input 32
GSM2797724	Input 33
GSM2797725	Input 34
GSM2797726	Input 35
GSM2797727	Input 36
GSM2797728	Input 37
GSM2797729	Input 38
GSM2797730	Input 39
GSM2797731	Input 40

Genome browser session
(e.g. UCSC)

no longer applicable

Methodology

Replicates

Duplicate experiments as described above and on ENCODE portal.

Sequencing depth	Each experiment >20M reads, single end 50, single end 75, single end 100, paired end 100. Details listed on ENCODE portal.
Antibodies	Listed above and on ENCODE portal.
Peak calling parameters	All settings described on ENCODE portal.
Data quality	All validation and QC are described on the ENCODE portal.
Software	Software listed above, described in the methods section of the manuscript, and on the ENCODE portal.
